# Development and optimization of an *in vivo* electrocardiogram recording method and analysis program for adult zebrafish

**DOI:** 10.1242/dmm.048827

**Published:** 2021-08-11

**Authors:** ThuyVy Duong, Rebecca Rose, Adriana Blazeski, Noah Fine, Courtney E. Woods, Joseph F. Thole, Nona Sotoodehnia, Elsayed Z. Soliman, Leslie Tung, Andrew S. McCallion, Dan E. Arking

**Affiliations:** 1 McKusick-Nathans Institute, Department of Genetic Medicine, Johns Hopkins University School of Medicine, Baltimore, MD 21205, USA; 2 Department of Biomedical Engineering, Johns Hopkins University, Baltimore, MD 21205, USA; 3 Cardiovascular Health Research Unit, Departments of Medicine and Epidemiology, University of Washington, Seattle, WA 98101, USA; 4 Epidemiological Cardiology Research Center (EPICARE), Wake Forest School of Medicine, Winston Salem, NC 27101, USA

**Keywords:** Zebrafish adults, Cardiac electrophysiology, Regression model development, Flecainide acetate

## Abstract

Clinically pertinent electrocardiogram (ECG) data from model systems, such as zebrafish, are crucial for illuminating factors contributing to human cardiac electrophysiological abnormalities and disease. Current zebrafish ECG collection strategies have not adequately addressed the consistent acquisition of high-quality traces or sources of phenotypic variation that could obscure data interpretation. Thus, we developed a novel platform to ensure high-quality recording of *in vivo* subdermal adult zebrafish ECGs and zebrafish ECG reading GUI (zERG), a program to acquire measurements from traces that commercial software cannot examine owing to erroneous peak calling. We evaluate normal ECG trait variation, revealing highly reproducible intervals and wave amplitude variation largely driven by recording artifacts, and identify sex and body size as potential confounders to PR, QRS and QT intervals. With this framework, we characterize the effect of the class I anti-arrhythmic drug flecainide acetate on adults, provide support for the impact of a Long QT syndrome model, and establish power calculations for this and other studies. These results highlight our pipeline as a robust approach to evaluate zebrafish models of human cardiac electrophysiological phenotypes.

## Abstract (precis)

**Summary:** We describe and establish an adult zebrafish electrocardiography acquisition and analysis strategy and evaluate its utility for examining zebrafish models of human cardiac electrophysiological phenotypes.

## INTRODUCTION

Cardiac electrophysiology involves the precise coordination of the electrical activity of the heart, which can be visualized and measured using the electrocardiogram (ECG). The waves in an ECG are a byproduct of atrial and ventricular depolarizations and repolarizations, and the intervals between the waves provide insight into the timing of these events. Although it is appreciated that alterations in cardiac electrophysiology are hallmarks and/or risk factors for a variety of human diseases (Ashar et al., 2018; Arking et al., 2014; Mizusawa and Wilde, 2012; Ntalla et al., 2020; Pflaumer et al., 2020; Schwartz et al., 2012; van der Harst et al., 2016), the processes that regulate it are less understood. We can begin to unravel the specific regulatory components by examining the basic biology underlying heart rate and cardiac electrophysiology to develop a framework to dissect related abnormalities. This has important clinical applications, because therapeutics targeting these regulatory components that modulate the electrical activity of the heart can be used as treatments for human diseases.

**Fig. 1.**
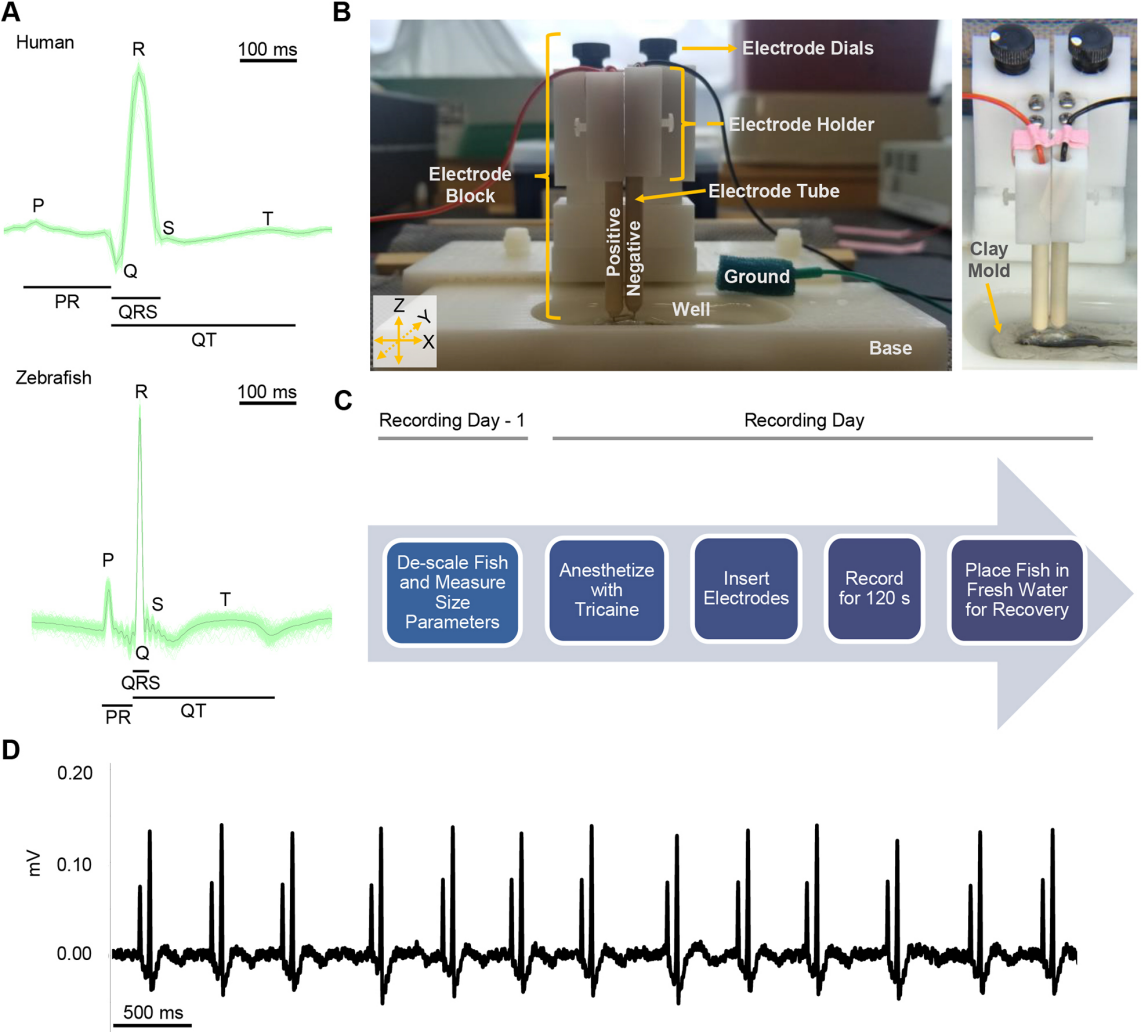
**Adult zebrafish ECG recording apparatus and protocol.** (A) Comparison of human and zebrafish ECG. Top: ECG of a healthy 32-year-old human male, obtained from the MIT-BIH Normal Sinus Rhythm Database (Goldberger et al., 2000). Bottom: ECG from a 6-month-old adult male AB zebrafish, captured using the described recording apparatus and protocol. All cycles within the 60 s (human) and 120 s (zebrafish) recordings were overlaid (green traces), and an average trace was calculated (black trace) using the LabChart ‘ECG Analysis’ module. Traces are shown on the same time scale for direct comparison. (B) Adult zebrafish ECG apparatus; during recordings, the zebrafish is placed ventral side up within the mold located in a well on the base. For ease of reference, apparatus components and axes used to describe the movement of the components are labeled. (C) Summary of adult zebrafish ECG recording protocol. (D) Example from a 6-month-old adult male AB zebrafish using the described recording apparatus and protocol. The trace image was captured using LabChart ‘Zoom View’.

The zebrafish (*Danio rerio*) is an excellent vertebrate experimental model system owing to its short generation time, low maintenance cost, large clutch sizes, ease with which it can be manipulated genetically (Irion et al., 2014; Moore et al., 2012; Varshney and Burgess, 2014), extensive developmental characterization and optical transparency (Gut et al., 2017). The zebrafish cardiovascular system includes a two-chambered heart (atrium and ventricle) and the bulbus arteriosus, a functional aorta surrogate structure (Vornanen and Hassinen, 2016). Although its two-chambered anatomy differs strikingly from that of the four-chambered human heart, zebrafish cardiac electrophysiology is comparable to that of the human cardiac system (Fig. 1A) (Goldberger et al., 2000). Notably, zebrafish resting heart rate [100-120 beats/min (bpm)] (Vornanen and Hassinen, 2016) is much closer to the human rate than that of traditional model organisms used to examine cardiac electrophysiology, such as mice (500-700 bpm) (Ho et al., 2011). Additionally, the zebrafish cardiac action potential (AP) is similar to the human cardiac AP. Although lacking the phase 1 repolarization stage, the zebrafish cardiac AP contains all other phases present in the human cardiac AP. Both the human and zebrafish ventricular APs feature a long plateau phase (Vornanen and Hassinen, 2016), as opposed to the mouse ventricular AP (Nemtsas et al., 2010). Examination of the AP from intact adult zebrafish atrium and ventricle revealed the existence of shared ion channels and currents that contribute to the cardiac AP in both organisms, notably atrium-only acetylcholine-activated K^+^ channels, L-type Ca^2+^ channels activated during the plateau phase, Na^+^ channels vital to the AP upstroke, and rapid delayed rectifier (I_Kr_) channels integral to repolarization (Nemtsas et al., 2010; Verkerk and Remme, 2012). Genetic similarities also exist; comparison of the human and zebrafish genomes has shown that 71% of human genes have at least one zebrafish ortholog (Howe et al., 2013). Overall, the high degree of similarity makes the zebrafish a suitable model organism to examine cardiac electrophysiology using ECGs.

The first adult zebrafish ECG recording method was developed in 2006 (Milan et al., 2006). Since then, several groups have developed additional methods and analysis software (Chaudhari et al., 2013; Lenning et al., 2017; Lin et al., 2018; Liu et al., 2016; Mersereau et al., 2015; Sun et al., 2009; Zhao et al., 2019). The core of these methods is the same, involving fish anesthetization and/or paralysis, electrode placement and/or insertion, followed by the capture of the ECG recording. However, there are many opportunities for the introduction of variation in data acquisition, processing and analysis that can obscure the signal within a study and lead to inconsistent findings across different studies. Such sources of variation can include the impact of the data acquisition date on trait recordings, variability inherent in the ECG recording method and the potentially confounding influence of biological/biometrical parameters exacerbated by the outbred nature of zebrafish. Current methods to capture ECGs in adult zebrafish do not incorporate methods to account for sources of variability in the acquired data.

Here, we present a framework for recording adult zebrafish ECGs that facilitates robust and reproducible acquisition of high-quality traces in a pipeline that is amenable to scale. We report 245 traces from 205 fish in different experimental conditions. Furthermore, we generate analysis software specifically built for examining zebrafish ECGs. Using this pipeline, we have identified significant covariates that should be considered for the study of adult zebrafish ECG traits, in addition to capturing cardiac electrophysiology phenotypes associated with flecainide acetate (FA) treatment and with a previously characterized Long QT syndrome zebrafish model, *kcnh6a^s290^* (Arnaout et al., 2007)*.* Our data demonstrate the robustness of our recording method and the need to account for biological confounders in the analysis of zebrafish ECG data to maximize their value in studying cardiac electrophysiology phenotypes pertinent to human disease.

## RESULTS

### Development of zebrafish ECG recording apparatus and protocol

Our experience with established ECG recording strategies highlights certain deficiencies. Initially, we used a system with non-invasive surface electrodes that resulted in a significant proportion of low-quality data with a large amount of noise. The recommended anesthetic agent, phenoxyethanol, minimized gill movement to improve trace quality but caused extremely low heart rates (<30 bpm) even in wild-type fish. Attempts to use another anesthetic (isoflurane-tricaine combination) led to heart rates closer to the norm, but trace quality suffered owing to increased gill movement. Within the literature, solutions to minimize gill movement and improve trace quality range from using neuromuscular blocking agents to performing open surgery to remove the membrane surrounding the heart. Although successful in the hands of others, we did not adopt these methods because they could introduce unnecessary difficulty and variation in the recording protocol. In transitioning to needle electrodes and attempting to adopt such methods described in the recent literature, we found that most do not allow for a high level of stability and consistency in terms of electrode placement (e.g. needles are inserted based on approximate distances that could differ between fish and/or the needles lack adequate support upon insertion).

To address these issues and refine the recording of ECGs in zebrafish, we adapted the PowerLab system, previously used for recording ECGs in mice and rats (Adriaens et al., 2018; Ha et al., 2020; Kim et al., 2019; Meo et al., 2016; Zhu et al., 2020). We integrated this system with components dissembled from the iWorx ECG system and fabricated components as follows. The commercial PowerLab system integrates a data acquisition (PowerLab) hardware device, which digitizes the ECG signals with an ECG signal amplifier (Animal BioAmp), processing the acquired data through the associated LabChart software. However, given that there is no standard PowerLab recording set-up for fish, we designed and fabricated a custom platform to facilitate ECG recordings (Fig. 1B). The platform consists of an ‘electrode block’, fabricated with dissembled components from iWorx. Two narrow electrode guiding tubes, positioned 5 mm apart, are attached to the electrode block. A 29-gauge needle electrode is placed through and projects ∼3 mm out from each tube; this length can be adjusted, if necessary. The electrodes can be lowered/raised independently using two dials on the electrode block. These dials allow adjustment of the height (*z*-axis) of the electrode holders to which each electrode tube is attached and their independent placement into contact with the zebrafish body. We adjust these holders and, consequently, the needle electrodes, such that ∼1-2 mm of the electrodes are inserted into the fish. Additionally, the position of the electrode block on the base can be adjusted to move on the *x*- and *y*-axes, allowing for increased control of electrode placement.

The platform is placed on top of a customized base, containing a well with a clay mold to hold the zebrafish during recordings, and is deep enough to hold ∼20 ml of liquid and is used to contain the anesthetic solution during recordings. The entire platform is housed inside a Faraday cage and on top of three vibration-absorbing mats to minimize mechanically induced artifacts that might introduce noise and compromise the quality of recorded data.

Using this customized system, we record ECGs in the 2 mV range at a rate of 2000 samples/s, using the following hardware filters: a low-pass filter at 200 Hz, a high-pass filter at 1 Hz and a mains filter; we refer to this as the ‘raw trace’. To reduce the amount of noise present in the traces further, a second tier of digital low-pass (100 Hz) and high-pass (3 Hz) filters are applied, and the resulting trace is used for all downstream analyses.

Within the 48 h before ECG recording, size parameters (length, width and weight) of the selected fish are documented. To facilitate electrode placement, fish are also descaled at that time, gently scraping the ventral surface at the level of the heart; this does not result in long- or short-term injury. The zebrafish are then separated and housed individually until the corresponding ECG is performed.

On the recording day, zebrafish are anesthetized with 0.643 mmol/l tricaine within the zebrafish facility (maintained at ∼30°C). Typically, a sufficient level of sedation is reached after 120-180 s, and immobility is confirmed by gently pinching the tail. After reaching the proper level of sedation, the fish is brought into the adjacent recording room and placed ventral side up in the mold within the well on the base that is filled with tricaine warmed to ∼30°C. The electrodes are then adjusted to hover over the fish by moving the entire electrode block on the *x*- and *y*-axes, using the dials on the base. When the electrodes are correctly positioned, with the aid of a magnifying glass lamp, electrode dials on the electrode block are adjusted to insert the positive (immediately anterior/cranial to the heart) and negative electrodes (caudal to the heart) slowly into the fish (Fig. S1). The ground electrode is placed into a piece of sponge and subsequently placed inferior to the zebrafish, ∼3-4 mm away from the edge of the well on the side closest to the electrode block; the sponge is oriented such that the start is parallel to the caudal fin and the end is parallel to the end of the well. To ensure that the trace reports the P wave and QRS complex cleanly when recording is initiated, the electrodes are adjusted [moving the electrode block, repositioning the electrode holders (*z*-axis) using the electrode dials, etc.]. The adjustment time is limited to ≤120 s; once this time has passed, the recording is captured. However, any irregularity or artifact in the waveform is noted in the recording manifest. The recording is captured for 180 s, with the last 120 s used for analysis. Traces that do not require adjustment or can be adjusted within the first 60 s have a total recording time of 180 s. Those that require 60-120 s for adjustment can have a total recording up to 300 s; although the recording during the adjustment time is saved, it is not considered part of the captured 180 s recording.

Once complete, the electrodes are removed, and the fish is returned to the fish facility and placed in a beaker with fresh system water (i.e. water from the zebrafish housing system) to recover before being returned into its respective tank. Tricaine in the well is discarded, and new, warm tricaine is prepared for the next fish. Fig. 1C summarizes our recording protocol, with an example trace in Fig. 1D. To assess the time required to execute our protocol, we examined time stamps from 179 traces recorded from experiments that did not require fish to be dosed in drug or vehicle control, because the time-sensitive nature of those experiments would inherently increase the duration of the protocol. Using this set of traces, the median protocol time per fish was determined to be 479 s (s.d., 113 s) (Fig. S2).

Before obtaining measurements, each trace is examined by an analyst, who is blinded to fish genotype or treatment received. The analyst assigns a quality score (1-5; best-worst) based on the distinct waveform and the amount of background noise present (Fig. S3); an additional score of 7 is assigned to traces in which the recording was halted owing to the inability to obtain a signal, even after adjustment. In our case, we proceed to examine only traces with a quality score of 1-4. Out of 245 recorded traces, 82% were graded as being of sufficient quality to obtain measurements based on quality score alone (Fig. S4).

### Development of custom zebrafish ECG analysis software

Our phase 1 analysis of all traces was performed with LabChart software using the ‘ECG Analysis’ module. However, 35% (70 of 200) of traces with quality scores ranging from 1 to 4 were not able to be analyzed, owing to the presence of a P wave with an amplitude that exceeded that of the R wave (Figs S4 and S5). LabChart incorrectly marks the P wave as the QRS complex, and, given that the ‘QRS max’ marker cannot be adjusted, these traces would ultimately be discarded. Although modulating parameters in LabChart could eventually allow these traces to be analyzed, no individual setting adjustment could compensate uniformly for this error. This imposed a time-consuming need for trace-specific adjustment and precluded the establishment of standardized parameters for analysis.

**Fig. 2.**
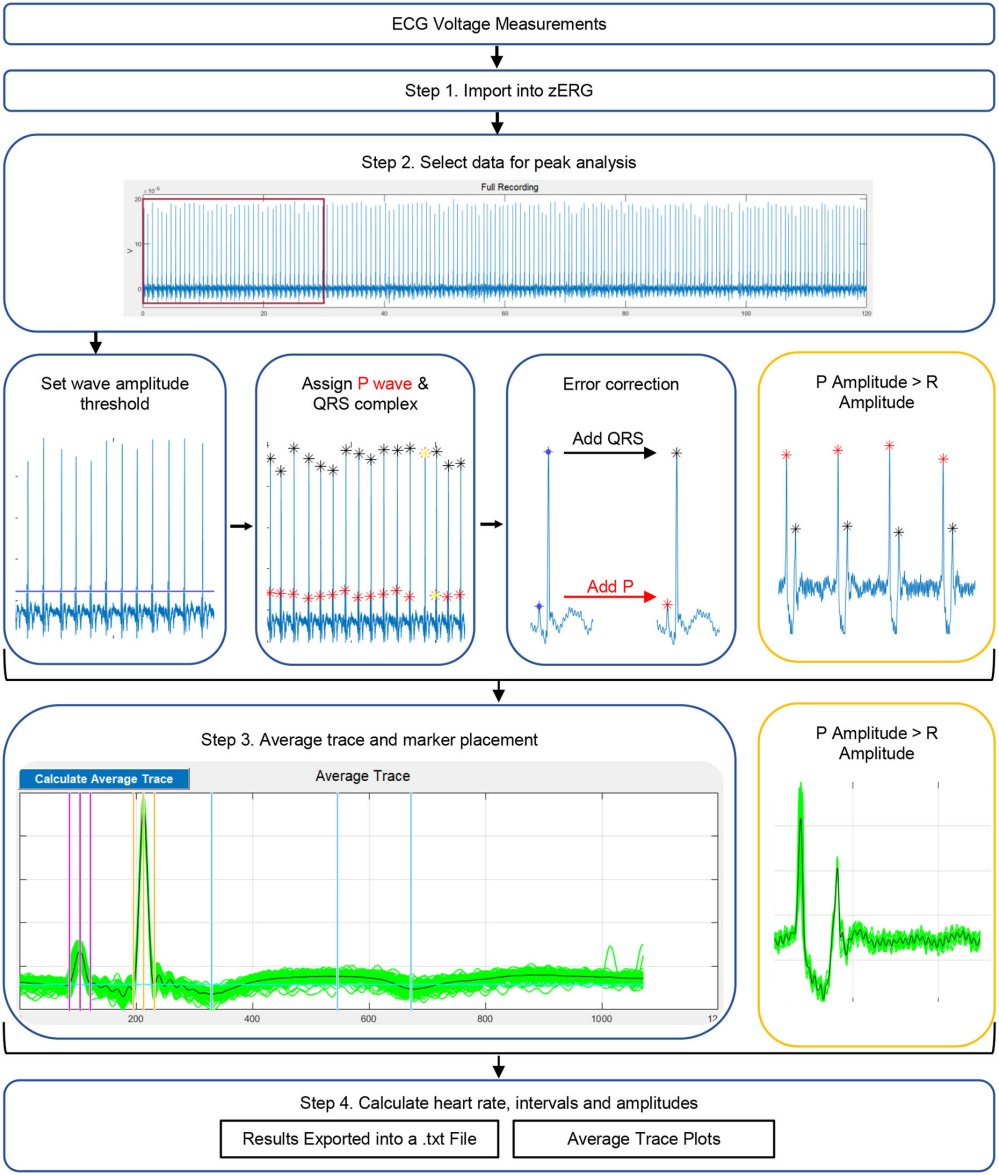
**Overview of zebrafish ECG reading GUI (zERG), a zebrafish ECG analysis software program.** In step 1, ECG voltage measurements are imported into zERG. In step 2, data for peak analysis are selected. During peak analysis, P waves (red asterisks) and QRS complexes (black asterisks) are identified based on the following: (1) the user-defined wave amplitude threshold; (2) the minimum peak distance; and (3) classification based on the distance between the P peak to R wave of consecutive cycles and the reference distance defined as two times the first ‘P peak to R wave’ interval. Consecutive red or black peaks are marked yellow for error correction; the user can then manually add and/or delete P waves and/or QRS complexes. Once all waves are marked correctly, the user can proceed to step 3 by choosing the ‘Calculate Average Trace’ button. This plots an average trace and allows the user to click on ‘Add Markers’ to note wave markers on the average trace. Finally, after all peaks are confirmed, the user selects ‘Analyze ECGs’ to obtain a graphical user interface (GUI) table with the ECG metrics, in addition to an output .txt and several average trace plots. The yellow boxes show an example of a trace in which the P wave amplitude exceeds the R wave amplitude and how zERG handles such traces. The example trace in the blue boxes was captured from a 6-month-old AB wild-type male fish, and the trace in the yellow boxes was captured from a 3.5-month-old AB wild-type male fish after being dosed with 0.800 mmol/l FA for 15* *min. All images were captured from zERG version 1.0.

To circumvent this limitation, we developed zebrafish ECG reading GUI (zERG) (Fig. 2) as a MATLAB-based graphical user interface (GUI) that can be used to analyze all traces, regardless of wave amplitude. The GUI is facile and accessible to those with the most rudimentary of computational experience. Voltage measurements are first exported from LabChart into a MATLAB data file (.mat), which is then imported into zERG; a textfile (.txt) containing voltage measurements and the corresponding times of these measurements can also be imported if a software package other than LabChart is used for ECG recordings. The user can choose to analyze the entire recording within the imported file or only a particular segment. After data selection, zERG uses the findpeaks algorithm to identify likely P waves and QRS complexes. The user selects the minimum peak height, for which peaks above this threshold and within an initial user-defined minimum peak interval are selected for analysis. To classify peaks as either P waves or QRS complexes, the following calculations are considered. In a given cycle, the interval between the P wave peak and the R wave is smaller than the interval from the R wave to the next P wave peak. The reference interval for the identification of all P waves and QRS complexes is considered as two times the first ‘P wave peak to R wave’ interval in the recording; that is, if the interval between any two given peaks is within this reference interval, the peaks are identified as a single cycle composed of one P wave and one QRS complex, regardless of wave amplitude. This component of zERG is essential for analysis of traces in which the P wave has a greater amplitude than the R wave and directly resolves the LabChart issues described above (Fig. 2, yellow boxes).

Once every wave in a trace is identified, the user can edit the automatic peak identification. Users can delete peaks called incorrectly and can add P waves or QRS complexes manually. Additionally, zERG highlights areas of the trace in which the algorithm has labeled peaks as consecutive P waves or QRS complexes, meaning that the user can easily identify and correct these potential errors. zERG also contains a noise-remover function, which can help the user to identify peaks more efficiently, particularly in traces in which a large proportion of noise is incorrectly called a P wave or QRS complex. Upon selecting the noise remover, the user is asked (1) to identify which peak has the greater amplitude and whether this relationship remains throughout the entire trace or whether there is a mixture; and (2) to identify the correct wave labeling within the first two cycles. Based on these answers, the noise remover automatically calls peaks using a combination of the minimum height of the taller peak, the interval between the P wave peak and R wave, and the interval of the R wave to the next P wave peak, but will ask for user input if it reaches a part where the parameters fall outside of what has already been defined. Incorrect peaks and places where the user stepped in are labeled for user review once the function is complete.

After all P waves and QRS complexes are called correctly, an average compiled trace is generated by aligning all marked QRS complexes; if necessary, the user also has the option to identify and align by the minimum in each ECG cycle. zERG includes several functions that allow modification of the display area before the P wave and/or after the QRS complex to be longer or shorter, in order to remove cycles that might contaminate the average compiled trace. Markers noting the start, maximum and end of the waves are then assigned. Once the markers are placed appropriately on the average compiled trace, the heart rate, RR, PR, QRS, QT intervals and wave amplitudes are calculated. zERG facilitates convenient export of these measurements into a tab-delimited .txt, which the user can then import into other software for statistical analyses. Interval and amplitude calculations are detailed in the Materials and Methods section, with reference buttons utilized in the zERG interface (labeled in Fig. S6).

The time for completion of trace analysis is quality dependent; the more noise in a trace, the more user time is spent in zERG correcting peak identification or providing more manual input during the noise-remover function. We tracked zERG analysis time for 182 traces and observed that higher-quality traces (scores 1 and 2) required significantly less time than lower-quality traces (scores 3 and 4) (Fig. S7). For a trace with minimal noise, an experienced user can obtain measurements within 60-180 s from the time of file import.

### zERG and LabChart yield concordant measurements

**Fig. 3.**
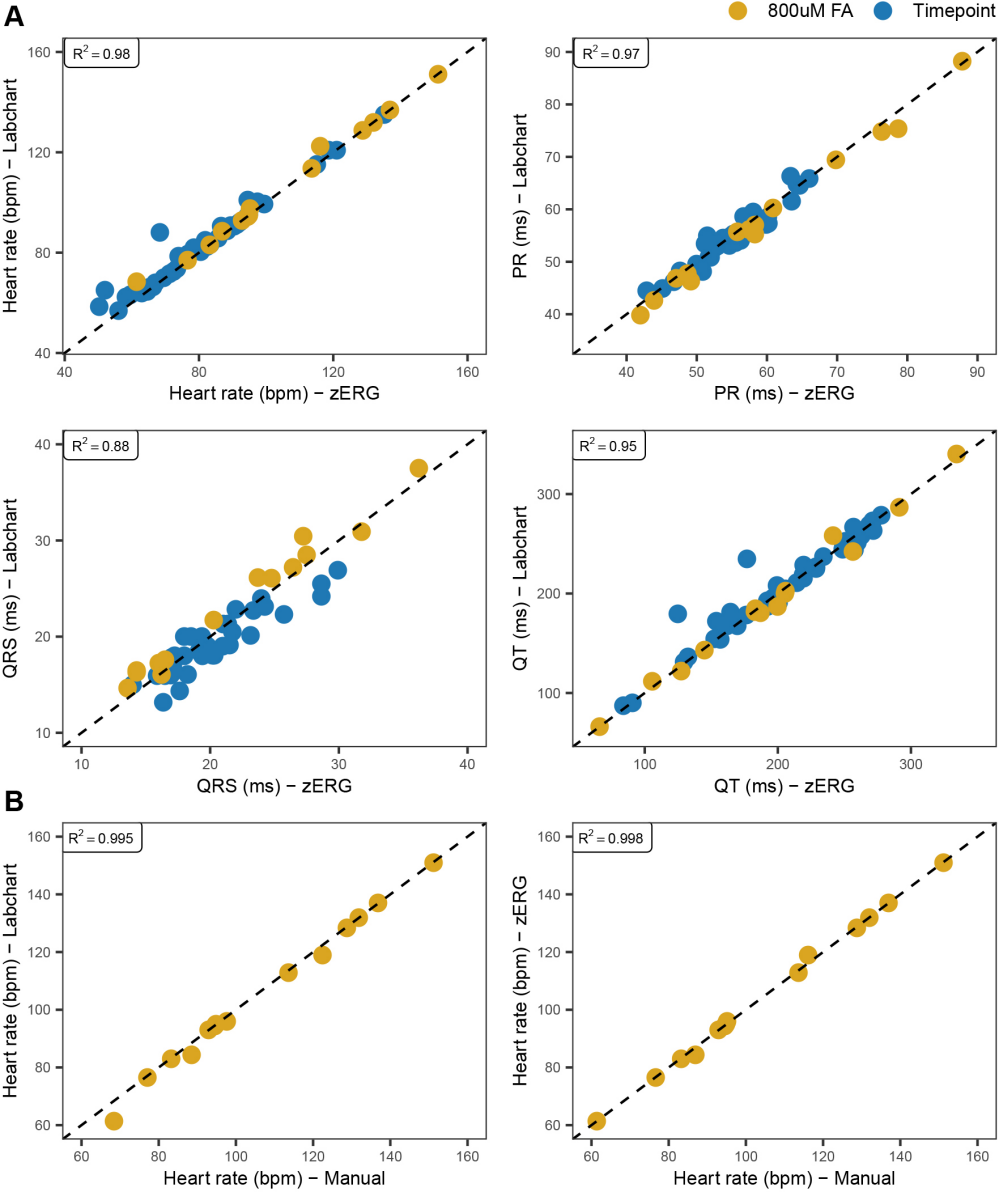
**Comparison of ECG metrics between zERG and LabChart.** Measurements from a total of 55 traces from two independent experiments, either from 0.800 mmol/l FA drug studies (*n*=14; referred to as FA) or from recording sessions in which 41 traces were captured from 11 AB fish over multiple timepoints (referred to as Timepoint), were used for comparison of zERG and LabChart performance. (A) Metrics obtained from zERG are nearly identical to those obtained from LabChart. (B) Comparison of heart rate between LabChart, zERG and a manual calculation. zERG heart rate calculations are nearly identical to those obtained from a manual calculation. bpm, beats/min.

To assess the performance of our custom ECG analysis software, we compared metrics obtained from zERG with those from LabChart. This necessitated a comparison of traces in which LabChart would not fail, i.e. where P wave amplitude did not exceed the R wave amplitude. Thus, we restricted our measurements to 55 traces for which both programs correctly identified peaks, using traces recorded from 11 AB wild-type fish at multiple timepoints and from FA drug studies. We observed that zERG generated measurements nearly identical to those from LabChart (Fig. 3A). Furthermore, we calculated heart rate manually for the subset of 14 FA traces to assess the accuracy of zERG heart rate calculations; manually calculated heart rates and those from zERG were also observed to be nearly identical (Fig. 3B). Unless otherwise stated, all analyses described below used zERG to obtain interval and amplitude measurements and include traces that LabChart would not handle appropriately.

### Characterizing the variability of zebrafish cardiac electrophysiology traits

Although several zebrafish ECG recording methods have been developed, little is known about the extent of normal intra-/interindividual variation present in zebrafish cardiac electrophysiology or the acquisition of trait data. One useful approach to examine the extent to which the trait data vary between normal fish is to record measurements from the same set of fish over multiple days. By doing so, we can examine, for each trait, the variance explained by two different terms: (1) the between-fish term, which accounts for how variable a trait is across a group of fish measured on a given day (Fig. S8A); and (2) the within-fish term, which stems from a combination of the variability of the measurement itself and that of the true biological signal (Fig. S8B). Owing to the inherent biological differences between zebrafish attributable to their outbred status, we expect that for any given ECG trait, the variance explained by the between-fish term would be greater than the variance explained by the within-fish term. By examining a ratio composed of the percentage of the total variance that is explained by the between-fish term to the percentage explained by the within-fish term, we can determine the variability of a measurement obtained from our recording system. Traits for which more variance is explained by the between-fish term than the within-fish term would be those we consider more reproducible and reliable.

To generate these ratios, we captured traces from 15 AB, 6-month-old wild-type fish on four consecutive days and analyzed the traces using zERG. Only traces that passed our quality score criteria and those from fish that survived the 4 day protocol were included; a total of 11 fish and 42 traces were used for this analysis. Using linear mixed models, we determined the variances explained by each term and the subsequent between-to-within ratio for heart rate, intervals and amplitudes (Table 1). For the amplitudes, very little of the variance was explained by the between-fish term in the model, suggesting that the signal is largely driven by recording artifacts. With respect to the intervals, PR had the largest ratio, indicating that it is the most reproducible interval measurement. To test whether the observed variance explained by the between-fish term could truly be attributed to measurements between fish, we permuted the fish identification variable randomly among the 42 traces such that traces were assigned randomly to different fish across the different days to generate a null model in which there was no difference in the variance explained by the between- and within-fish terms. Under the null model, the between-fish term no longer explained a large proportion of the variance and the between-to-within ratio decreased for the intervals, indicating that the variance explained by the between-fish term is attributed primarily to that of the inherent and expected biological differences between AB wild-type fish.

**Table 1.**
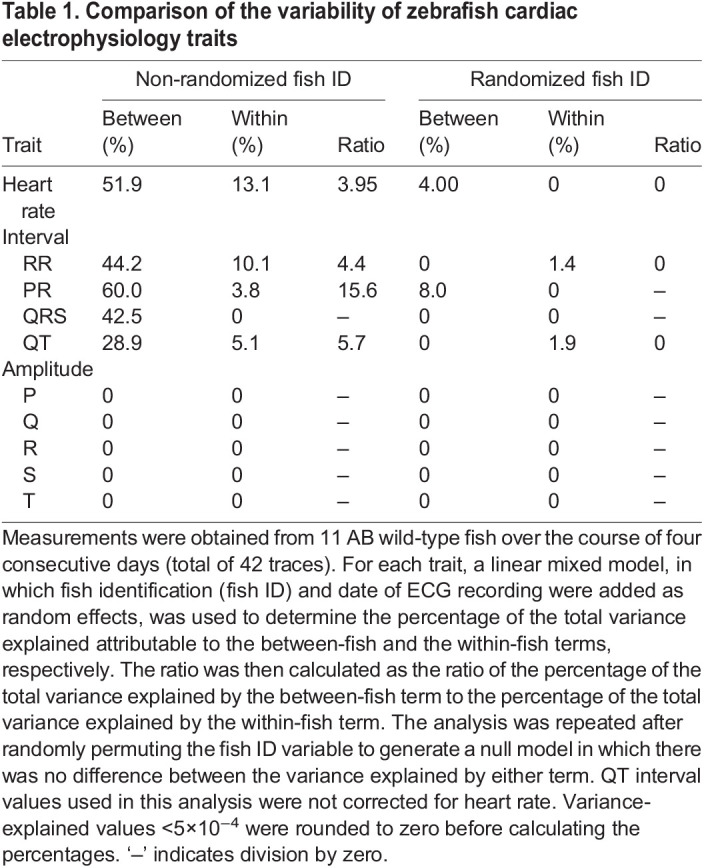
Comparison of the variability of zebrafish cardiac electrophysiology traits

### Defining correlation among zebrafish cardiac electrophysiology traits

**Fig. 4.**
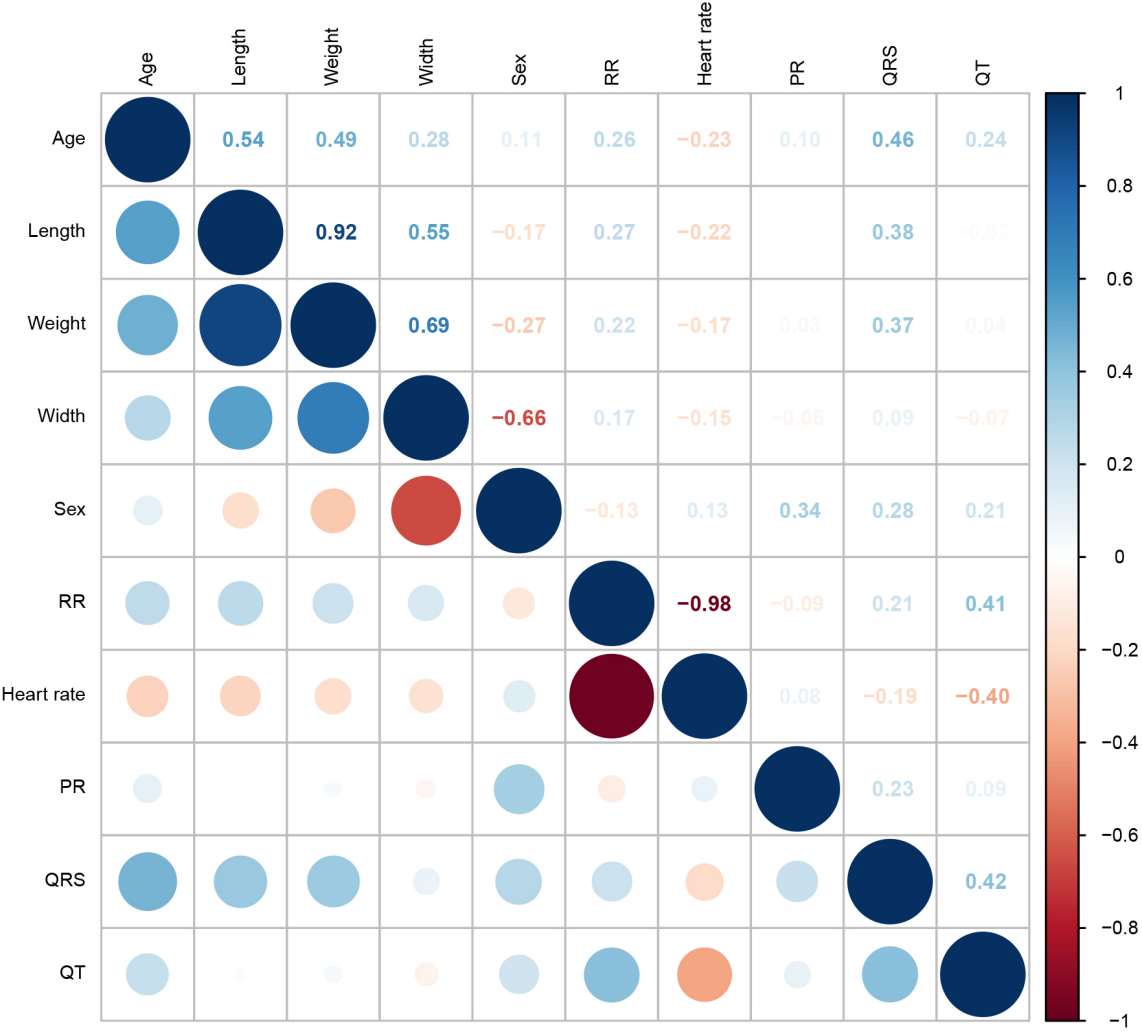
**Correlation of zebrafish ECG traits and covariates.** Zebrafish ECG traits (heart rate and intervals) are correlated with each other and with body size parameters, sex and age. The correlation matrix is derived from measurements obtained from a total of 70 AB and wild-type fish from the *kcnh6a^s290^* and *kcnh6a^tb218^* lines. Positive correlations are colored in blue and negative correlations in red. The color intensity and size of the circles are proportional to the correlation (i.e. a correlation of 0.80 will have a larger and darker red circle than a correlation of 0.10).

We next identified variables that might serve as potential confounders in the analysis of zebrafish cardiac electrophysiology traits using traces recorded from a total of 70 wild-type fish ∼3-6 months old. We combined both AB (including the first measurement of fish where we captured traces over multiple timepoints) and wild-type fish from two different lines: *kcnh6a^s290^* and *kcnh6a^tb218^*. Traces were captured during ECG recording sessions held on different days and at two separate recording locations. Table S1 lists the average values (mean±s.d.) for heart rate and the intervals from these 70 fish. Correlation analysis from this dataset revealed correlation among ECG traits and with body size parameters (length, width and weight), sex and age (Fig. 4). Additionally, heart rate was negatively correlated with QT interval (*r*=−0.40).

### Identifying potential confounders in zebrafish cardiac electrophysiology traits

In parallel with the correlation analysis, we used linear regression models to identify covariates that should be included in ECG trait analyses. For this analysis, we used the same dataset as already described for the correlation analysis. To correct for batch effects, we initially used a linear mixed model to obtain residuals for each trait (heart rate and intervals) after adjusting for age and recording location (fixed effects) and the date of the recording (random effect). We then used these residuals for model selection based on Akaike information criterion (AIC) values using a forward regression approach. Table S2 lists the terms included, for each trait, in the model that produced the lowest AIC value. Sex was included in all traits, and, as expected, the model for QT included heart rate. Although both weight and length were included in the QT model, the correlation analysis indicated that these body size parameters were highly correlated (*r*=0.92). Based on these results, all analyses described below used either a linear regression or a linear mixed model that included sex and weight as covariates, with heart rate as an additional covariate for QT, unless otherwise noted.

### zERG reveals PR and QRS elongation in FA-treated adult zebrafish

The class I anti-arrhythmic drug, FA, is used to treat atrial fibrillation and tachyarrhythmias in humans and is known to increase cardiac electrophysiological parameters (PR, QRS and QT) (Echt and Ruskin, 2020). Previous research showed that FA treatment affected embryonic zebrafish cardiac electrophysiology (Chopra et al., 2010). However, the impact of FA on zebrafish adults is largely unknown. Using our ECG system and analysis software, we examined the effect of FA treatment on adult zebrafish cardiac electrophysiology.

**Fig. 5.**
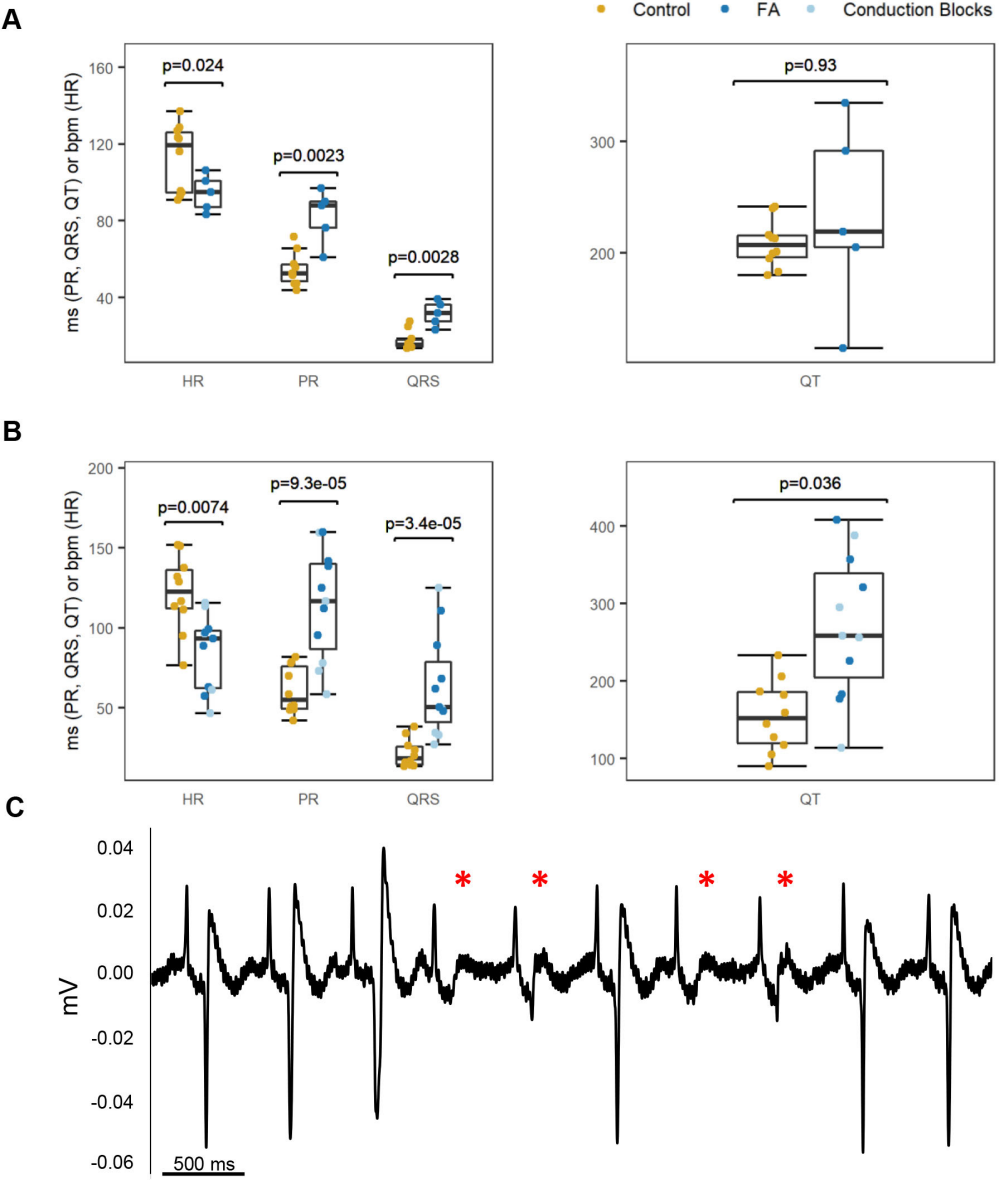
**Impact of 0.800 mmol/l FA treatment on adult zebrafish cardiac electrophysiology.** (A) Measurements were acquired from 15 adult zebrafish (5.2* *months old) dosed for 30* *min in either DMSO vehicle control (*n*=10) or FA (*n*=5). Compared with controls, FA-dosed fish displayed a slower heart rate (*P*=0.024) and significant prolongation of PR (*P*=0.0023) and QRS (*P*=0.0028) intervals. (B) Measurements were obtained from 21 adult zebrafish (3.5* *months old) dosed for 15* *min in either DMSO vehicle control (*n*=10) or FA (*n*=11). In addition to a significant decrease in heart rate (*P*=0.0074) and prolongation of PR (*P*=9.3×10^−5^) and QRS (*P*=3.4×10^−5^) intervals, FA-dosed fish in this experiment also displayed significant QT interval prolongation (*P*=0.036) compared with control fish. Several FA-dosed fish (*n*=6) within this independent experiment experienced conduction blocks (light blue points in B). The *P*-values in A and B were determined from a linear regression adjusting for sex and weight; QT interval was additionally adjusted for heart rate. QT interval plots show the measurement uncorrected for heart rate. (C) Representative trace from a 3.5-month-old male fish dosed with FA for 15* *min; the red asterisks denote conduction blocks, as evidenced by the conduction failure (no QRS complex). The trace image was captured using LabChart ‘Zoom View.’

We dosed AB fish for ≤60* *min with several FA and dimethyl sulfoxide (DMSO) vehicle control concentrations to observe the dose response and determine an appropriate dose range (Tables S3 and S4). We observed erratic swimming behavior and/or mortality at three different FA doses (0.250, 0.500 and 0.731 mmol/l). We used the highest dose as a benchmark, because we desired a dose that would be most likely to result in a potential phenotype. For this reason, we treated 5.2-month-old wild-type AB zebrafish with 0.800 mmol/l FA for 30* *min. Measurements were acquired from a total of 15 fish by analysts who were blinded with respect to which treatment (drug or 0.95% DMSO control) a fish received. Consistent with the expected drug activity, we observed a significant prolongation of PR and QRS intervals, in addition to slower heart rates, in FA-dosed fish compared with control fish (Fig. 5A). We also noted a difference in mortality between groups, where time of death was defined as any time from the end of the recording session to time in recovery. Although none of the fish died during the recording, three of five FA-dosed fish did not recover from the protocol, as opposed to all ten of ten control fish that recovered and survived.

To examine whether this effect remains when dosing time is decreased and to generate an overall larger sample size (total of 21), we dosed 3.5-month-old wild-type AB zebrafish with 0.800 mmol/l FA or DMSO vehicle control for 15* *min. Again, we observed a significant prolongation of PR and QRS intervals in addition to slower heart rates in FA-dosed fish compared with control fish. FA-dosed fish also displayed significant QT interval prolongation compared with controls (Fig. 5B). Using the same definition of mortality, all ten control fish survived, as opposed to one of 11 FA-dosed fish. Additionally, six of 11 FA-dosed fish compared with zero of ten controls suffered conduction blocks (Fig. 5C), as evidenced by the failure of the QRS to appear in the cycles indicated by the red asterisks; note that the P wave morphology does not change, which is consistent with a temporally downstream effect. Taken together, these results indicate that our system can capture cardiac-relevant phenotypes induced by treatment with drugs known to perturb cardiac electrophysiology.

### Delayed ventricular repolarization in *kcnh6a^s290^*^/+^ mutants

**Fig. 6.**
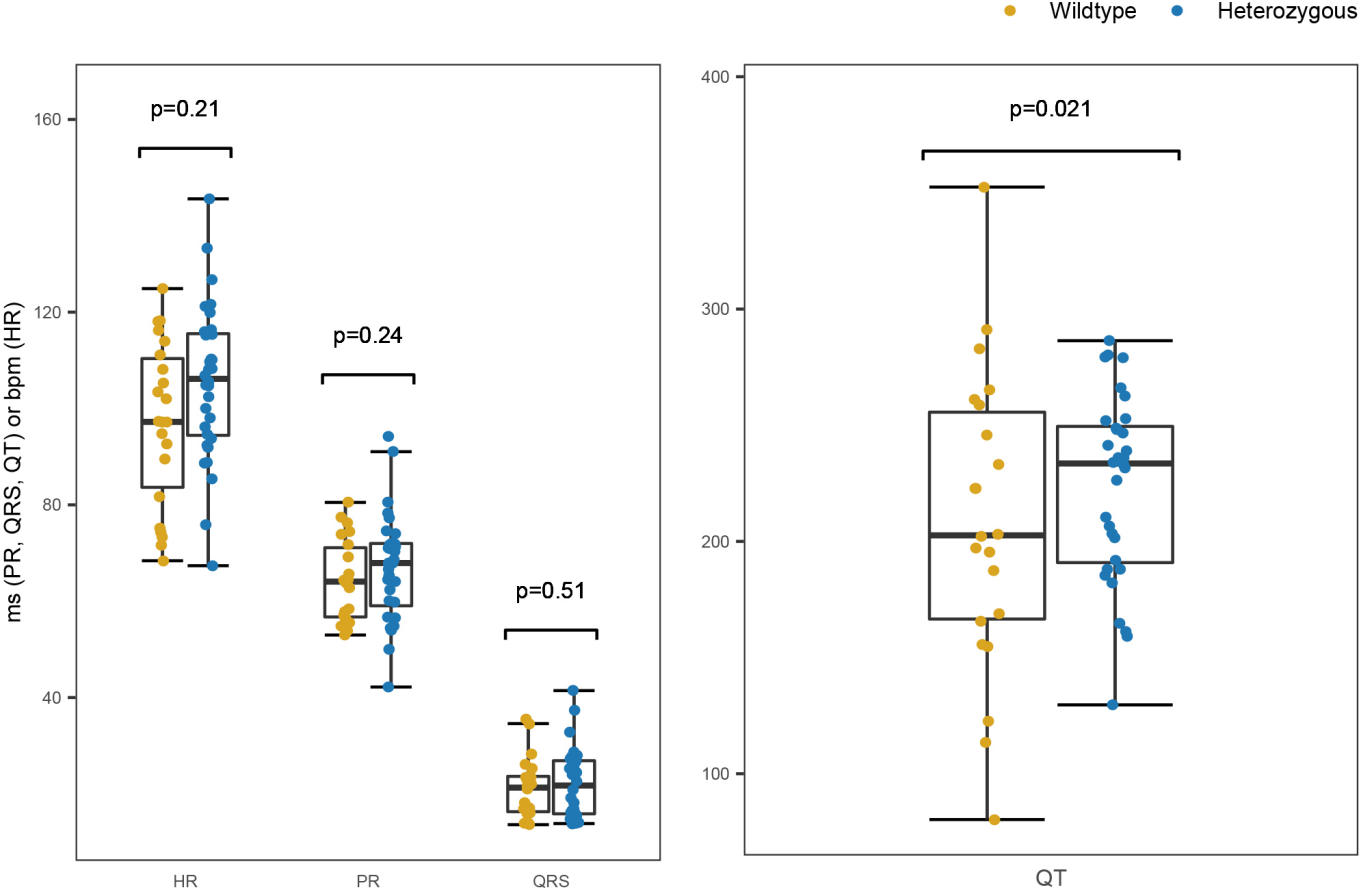
**Delayed ventricular repolarization in *kcnh6a^s290^*^/+^ mutants.***kcnh6a^s290^*^/+^ (heterozygous) mutants displayed a longer QT interval than their *kcnh6a*^+/+^ (wild-type) clutchmates (*P*=0.021). Measurements were obtained from a total of 54 fish. The *P*-values were determined from a linear mixed model, in which the date of the ECG recording session was added as a random effect and age, ECG recording location, sex and weight were added as fixed effects; QT interval was additionally adjusted for heart rate. The QT interval plot shows the measurement uncorrected for heart rate.

Given the large effects observed with the FA doses, we sought to validate the utility of our ECG protocol further by testing a more subtle phenotype. The zebrafish mutant line *kcnh6a^s290^* has been characterized as a model for Long QT syndrome; adult *kcnh6a^s290^*^/+^ zebrafish were reported to have an increased QT interval, suggesting dysfunction of ventricular repolarization (Arnaout et al., 2007). We captured traces from a total of 54 fish ranging from 3.7 to 6.2 months old (*kcnh6a*^+/+^, *n*=22; *kcnh6a^s290^*^/+^, *n*=32) over three separate recording sessions; analysts were blinded to genotype, and the recording order for each recording session was randomized. We observed no differences in heart rate or cardiac conduction but a significant prolongation of QT interval in the *kcnh6a^s290^*^/+^ group compared with their wild-type clutchmates (Fig. 6), consistent with previous observations (Arnaout et al., 2007).

### Adult zebrafish ECG trait power calculations

Finally, in developing another tool to aid in the body of zebrafish research, we performed power calculations using the data collected from a total of 70 AB and wild-type fish from the *kcnh6a^s290^* and *kcnh6a^tb218^* lines. We first adjusted the ECG traits using a linear mixed model to obtain residuals, and we included the following covariates: ECG recording date (random effect) and location of ECG recording, age, sex and weight (with QT interval additionally adjusted for heart rate), all as fixed effects. Using the residuals and raw trait means, we determined the sample size (per group) required to detect various percentage differences in means. Table 2 highlights our results. We use PR interval as an example of our calculation. Based on our studies, the mean and residual standard deviation for AB wild-type fish PR is 58.7 ms and 7.63 ms, respectively. Therefore, for 80% power to detect a 10% difference in the means between two groups, our data suggest that 25 fish per group are required at a significance level of 0.05.

**Table 2.**
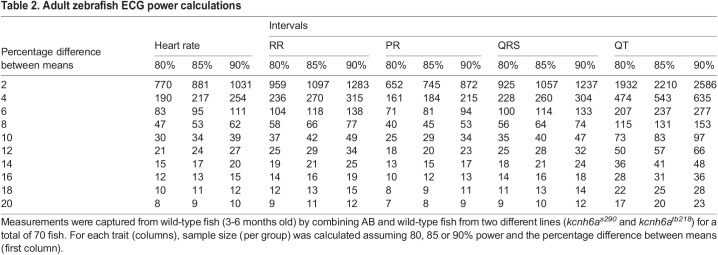
Adult zebrafish ECG power calculations

## DISCUSSION

We developed an *in vivo* adult zebrafish ECG system that allows the rapid capture of high-quality traces, and established an analytical platform that accounts for variance in and confounders of the data and facilitates determination of appropriate study size through biologically informed power calculations. Although several adult zebrafish ECG systems have been devised (Chaudhari et al., 2013; Lenning et al., 2017; Lin et al., 2018; Liu et al., 2016; Mersereau et al., 2015; Milan et al., 2006; Sun et al., 2009), none to our knowledge has afforded the kind of throughput and statistical rigor reported here. Our data highlight the novelty of zERG and the advantages it provides in maximizing usable traces, in addition to the ease and rigor it provides the user in analyses.

In the development of this system, we recorded 245 traces from 205 fish, of which 82% of traces were of sufficient quality to obtain reliable measurements. Our experimental framework requires minimal preparation and recording time and allows the analysis of ≤50 fish within a working day. Although one recent study (Zhao et al., 2019) uses the PowerLab equipment from which we borrow components and Labchart software, their method relies on extensive electrode adjustment. One may assume this is driven by the observation that needle electrode placement, particularly in the pectoralis musculature, could cause the P wave amplitude to be larger than the R wave amplitude, leading LabChart to misidentify the P wave as the QRS complex and thus losing valuable traces from analysis. Controlled, consistent electrode placement is vital in generating good-quality ECG traces. Our customized apparatus is designed such that the distance between the electrodes and the depth to which they are inserted are relatively fixed across all fish. Furthermore, our custom fabricated platform allows adjustment of the electrode block or depth of tissue penetration with *x*-, *y*- and *z*-axis dialed manipulation. In many traces in which the P wave amplitude exceeded the R wave amplitude, we were able to adjust the electrode placement incrementally and correct it such that the R wave amplitude exceeded the P wave amplitude, and they were then detected appropriately.

However, 30% of all recorded traces (regardless of QC score) could not be resolved in this manner, or the resulting trace contained a subset of cycles in which the P wave amplitude was greater than the R wave amplitude. In either case, analysis using LabChart incorrectly identifies the waves, resulting in production of an erroneous average trace. As an example of this burden, only eight (five controls, three FA) and seven (six controls, one FA) traces from the 30 min and 15 min FA dosing experiments, respectively, were eligible for LabChart analysis; relying solely on this commercial software would have resulted in the discarding of >50% of otherwise usable data. This severe limitation motivated us to develop zERG. We demonstrate that zERG defines non-problematic traces as efficiently as LabChart but provides a spectrum of advantage over the commercial counterpart. Not least of these advantages is the capacity to define P waves systematically and correctly even in previously problematic traces, meaning that otherwise intractable data are no longer lost to analyses. Furthermore, zERG flags potential errors in peak identification and renders them amenable to correction (under defined rules) and provides these tools in a user-friendly GUI on the established MATLAB platform. The data arising from zERG are thus amenable to a wide array of statistical tests to determine the significance of our observations.

Importantly, zebrafish present the additional genetic complication of being a non-inbred system. To our knowledge, no reports acquiring zebrafish ECGs have evaluated the day-to-day natural variation in traits, e.g. by recording traces from the same set of zebrafish over multiple consecutive days. Implementing a pipeline of linear mixed models, we are able to demonstrate that the study of zebrafish ECGs should focus on examining the intervals observed in traces rather than comparison of amplitude. We show the latter to be more indicative of technical artifacts, such as electrode placement, rather than true biological signal. Among our interval analysis, our data reveal that PR is the most reliably reproducible trait. Exporting high-quality ECG data into a model-based statistical framework allows us to determine whether analyses of zebrafish ECG data should account for the potential for confounding effects that could obscure or erroneously provide signal.

Extending these observations, using measurements from a total of 70 wild-type fish at 3-6 months of age, we investigated the relationship between zebrafish ECG traits and body size parameters, age and sex. Our data reveal sex and age differences in zebrafish ECG traits. Although not strong, all ECG traits are positively correlated with female status, with PR exhibiting the strongest correlation. Age is positively correlated with QRS and QT interval and negatively correlated with heart rate. The observed correlation between heart rate and QT interval is consistent with observations in humans, although not as strong (−0.40 versus −0.76 in middle-aged adults; Soliman and Rautaharju, 2012). These results provide evidence reinforcing the utility of zebrafish as models for examining cardiac electrophysiology to the study of human disease. They also highlight the need to account for previously undescribed confounders in the study of zebrafish cardiac electrophysiology.

Using the devised recording system and set-up, we tested the effect of FA on adult zebrafish and determined that FA treatment for 30* *min resulted in increased PR and QRS intervals and a decrease in heart rate. Upon increasing our overall sample size, we recapitulated these results and observed a significant effect on QT interval despite reducing the dosing time to 15* *min. These observations agree with our expectations based on the effect of FA on human cardiac electrophysiology and are consistent with the expected activity of a class Ic anti-arrhythmia drug (Echt and Ruskin, 2020). Within the 15 min dosing recording session, several FA-dosed fish suffered from conduction blocks. This was observed in data from Chopra et al. (2010), albeit in embryos. They reported that all 45 embryos reared in 0.400 mmol/l FA from the 64-cell stage to 24-48 h (or occasionally 60 h) post-fertilization experienced 2:1 heart block and bradycardia. Notably, we did not observe conduction blocks in data from dosing 5.2-month-old wild-type AB zebrafish with 0.800 mmol/l FA for 30* *min. Whether this is a function of age or drug acclimation owing to the increased FA dosing time requires additional study.

We then asked whether our strategy could go beyond characterizing chemically induced perturbations of conductance to characterize a clinically relevant conductance phenotype correctly. To this end, we captured traces from a previously characterized Long QT syndrome *kcnh6a^s290^* mutant zebrafish line reported to display delayed ventricular repolarization. Despite the observation that T waves can occasionally be challenging to identify, we evaluated QT interval measurements successfully in this mutant line. The resulting data provide a statistically robust demonstration of prolonged QT intervals in *kcnh6a^s290^* mutant fish compared with their *kcnh6a* wild-type clutchmates.

Compared with the FA experiments, this phenotype is more subtle. Over the past few years, the number of zebrafish models generated for studying human disease related to cardiac electrophysiology has increased (Gut et al., 2017; Verkerk and Remme, 2012; Vornanen and Hassinen, 2016). Zebrafish are a powerful model system, but to improve on their utility it is vital to develop phenotyping tools and strategies that will aid in experimental design to take full advantage of such a system. Importantly, we described power calculations to determine the study size necessary to evaluate a range of ECG trait effect sizes, using measures of variance observed in traces collected from a total of 70 AB and wild-type fish. In sum, our results demonstrate the utility and applicability of our method and analysis framework to study zebrafish models of a spectrum of human cardiac electrophysiology phenotypes.

Despite similarities between human and zebrafish cardiac electrophysiology, it is imperative to recall differences to understand the caveats of studying zebrafish ECGs. Morphologically, the zebrafish heart consists of only two chambers. The zebrafish cardiac AP lacks the fast phase 1 repolarization present in the human cardiac AP, despite having a similar shape overall. Ion channel composition within cells can differ, such as the presence of T- and L-type calcium currents in zebrafish atrial and ventricular myocytes as opposed to their presence only in human sinoatrial node and conductive tissues (Nemtsas et al., 2010). Zebrafish heart rate and cardiac APs can be temperature dependent (Lee et al., 2016; Lin et al., 2014; Vornanen, 2016) and are generally measured at lower temperatures than human cardiac APs (which become prolonged at these temperatures) (Verkerk and Remme, 2012). There are also differences in ion current generation and maintenance. As an example, the human I_Kr_ is generated by *KCNH2*, whereas the zebrafish current is generated by the zebrafish ortholog to the mammalian *KCNH6* (Vornanen and Hassinen, 2016). These caveats illustrate the importance of careful experimental design and data interpretation to ensure the appropriate translation of findings in zebrafish to human cardiac electrophysiology.

Our system offers notable advances, but we recognize several study limitations. First, the majority of fish analyzed were 2.7-6.2 months old. Other groups have generally focused on older fish, 10-12 months old (Lin et al., 2018; Liu et al., 2016; Zhao et al., 2019). It would be prudent to examine older fish to see whether we observe the same correlations and subsequent models as seen in younger fish. Particularly, given that our results suggest an effect of aging on zebrafish cardiac electrophysiology, it would be interesting to observe whether aging affects the severity of the phenotype in mutant zebrafish lines of genes believed to affect cardiac electrophysiology. Second, we capture traces for a short time (180-300 s) to maximize throughput and limit concern regarding prolonged use of anesthesia. Although we can record ≤50 fish per working day, we recognize that our system would not allow for throughput work at the level of hundreds of fish per day unless there are significant redesigns, such as faster and automated manipulation and placement of electrodes with robotics and/or micromanipulators, decreasing recording time and/or time to induce sufficient anesthesia. With regard to tricaine, it is well known that tricaine can decrease heart rate over a period of time (Huang et al., 2010; Martins et al., 2018). Although other anesthetics have been suggested (tricaine combined with isoflurane, ketamine, 2-phenoxyethanol, etc.), tricaine is the only US Food and Drug Administration (FDA)-approved fish anesthetic. Third, as previously mentioned, zebrafish heart rate and cardiac APs are temperature dependent, but we have yet to examine the impact of temperature variation on our protocol.

Despite its robustness and ease of use, zERG has several programming limitations. Although an efficient and simple program, it is currently not designed to detect and identify arrhythmias automatically. As an example, zERG will recognize conduction blocks as an anomaly and note them with two consecutive, overlaid red and yellow markers. However, it cannot identify the anomaly as a conduction block; this burden still falls on the user. Second, although the majority of analyzed traces required <5 min to examine, several required the analyst to spend >10 min per trace. These traces either contained a large number of trace artifacts or exhibited unusual QRS waveforms requiring manual identification and correction of the P and R waves. We are actively working to improve our wave detection algorithms to decrease trace analysis time and allow for uncommon waveforms to be analyzed efficiently.

In conclusion, we refined and validated a zebrafish ECG recording system and analysis software package that allows for the efficient collection of high-quality traces from ≤50 fish within a working day. We provide straightforward statistical recommendations to determine and regress away the influence of confounding effects on our analyses and, in doing so, provide a framework in which it is possible to anticipate the study size necessary to make statistically robust observations and, ultimately, an efficient strategy for the acquisition, processing and analysis of zebrafish ECGs. In examining zebrafish cardiac electrophysiology, we can begin to unravel crucial components regulating heart rate, in addition to atrial and ventricular depolarizations and repolarizations, and apply these findings toward understanding human disease.

## MATERIALS AND METHODS

### Zebrafish maintenance

Adult AB, *kcnh6a^s290^* and *kcnh6a^tb218^* mutant zebrafish lines were maintained in system water according to standard methods (Westerfield, 2007) at the Johns Hopkins University School of Medicine East Baltimore campus. All zebrafish experimental procedures were performed in accordance with the ethical permits set by the Johns Hopkins Institutional Animal Care and Use Committee.

Acquisition of the *kcnh6a^s290^* line was obtained from Martin Tristani-Firouzi's group at the University of Utah as embryos from heterozygous/heterozygous crosses. The *kcnh6a^tb218^* line was brought in from David Milan's group (Cardiovascular Research Center, Massachusetts General Hospital, Boston, MA, USA) as embryos from heterozygous crosses. Adult fish from each mutant line were crossed to AB fish taken from our own facility for several generations before progeny were crossed appropriately and used for subsequent analyses.

All adult zebrafish used for experiments were from the AB strain and were in the age range of 2.7-6.2 months, unless otherwise stated. Adult zebrafish of both sexes were used for all experiments. The distribution of females and males used is noted in the figure legends and under each section detailing methods for each respective experiment within the Materials and Methods.

### Genotyping

Genotyping of both zebrafish mutant lines was completed using PCR and an enzyme digestion assay. DNA was extracted from a 3 mm section of tail fin clipping using 50 µl lysis buffer (1 ml of 1 M Tris-HCl at pH 8.4, 5 ml of 1 M KCl, 150 µl of 1 M MgCl_2_, 1.5 ml 20% Tween-20, 3 ml 10% NP-40 and 89.35 ml deionized water) incubated at 95°C for 20* *min. Then, 5 µl proteinase K (03115828001; Roche) was added to the lysis buffer and incubated at 55°C for 1 h; the reaction was inactivated by incubating at 95°C for 20* *min.

The *kcnh6a^s290^* line is identified by a T>A point mutation. Primers s290 kcnh6 MUT T7 F1 (5′-TAATACGACTCACTATAGGGAGCCAGAACAGAACGAAACG-3′) and s290 kcnh6 MUT T7 R1 (5′-ACCCCAGTGTTTTGAATGGT-3′) were designed to flank the site of the point mutation for a total product size of 753 bp. To amplify this site, 5 µl GoTaq Green Master Mix (M7123; Promega Corporation) was mixed with 0.625 µl of 10 µM upstream primer, 0.625 µl of 10 µM downstream primer, 2 µl genomic DNA and 21.75 µl nuclease-free water, for a total reaction volume of 50 µl. The PCR conditions were set as follows: an initial denaturing step was run for 2 min at 95°C followed by 35 cycles of 30 s of denaturation at 95°C, 30 s of annealing at 60°C and 30 s of extension at 72°C. An additional 10 min of extension at 72°C was done after the final cycle was complete. Following PCR, 10 µl of the PCR product was combined with 1 µl restriction enzyme smlI (R0597L; New England Biolabs), 3 µl CutSmart 10× Buffer (B7204S; New England Biolabs) and 16 µl nuclease-free water for a 30 µl reaction. This reaction was incubated at 55°C for 1 h before being run on a 2% agarose gel. Results from this enzyme digestion will show cut sites at 405 bp and 348 bp only when the wild-type sequence is present.

A similar assay was used to genotype *kcnh6a^tb218^*. Following the same PCR mix and cycling conditions as listed above, we used primers tb218 T7 geno F3 (5′-TAATACGACTCACTATAGGGTTGGTGGGTGAGGCTAAAGA-3′) and tb219 T7 geno R3 (5′-ATGCACTGGGTCTCTGCAA-3′) to amplify an 853 bp region around a T>G point mutation. After PCR, a 30 µl reaction was set up using the restriction enzyme sphI (R3182S; New England Biolabs). This reaction was incubated at 37°C for 1* *h, followed by 20* *min of heat inactivation at 65°C. Results from this enzyme reaction will show a 566* *bp band when the wild-type sequence is present, whereas a 292* *bp band appears if the mutant sequence is present.

### Design and assembly of zebrafish ECG apparatus

Assembly of the ECG recording apparatus was completed by the Johns Hopkins University Center for Neuroscience Research. The apparatus is composed of two main parts: a base containing an oval well to hold ∼20 ml liquid (major axis diameter, 6.35 cm; minor axis diameter, 2.54 cm) and an electrode block that comes from dismantling the iWorx Zebrafish ECG Recording (ZS-100; iWorx). The dimensions of the entire base are 12.7×10.16×0.8636 cm. The base is divided further into two components: a static front portion (dimensions, 12.7×4.445×1.905 cm) that contains the well and a back portion that can be adjusted to move on the *x*-axis. The dimensions for this back component are as follows: 12.7×5.715×0.9525 cm. Two screws (diameter, 0.635 cm) along their individual 5.715 cm slit allow for this back component to be moved. After dismantling the iWorx component, it was adhered onto the center of the base on top of a rectangular block (dimensions, 5.08×1.905×1.27 cm). The positive and negative 29-gauge needle electrodes (MLA1204; ADInstruments) are inserted through two narrow tubes (diameter, 0.4826 cm; length, 5.8674 cm) that are held to hover over the well by two individual electrode holders (dimensions, 0.7874×2.54×2.54 cm). Two screws (diameter, 0.4826 cm) connected to the side of the electrode holders are used to tighten the electrode tube within the holders. The electrode tubes extend from the electrode block and down into the well, with the needles placed directly above the well containing the clay mold and extending ∼1-2 mm out from the tube. The electrode block can be moved on the *y*-axis via a component that the electrode block sits upon and extends out of the base, similar to the shape of forklift forks; these forks have the following dimensions: 5.08×7.62×0.635 cm.

All parts, with the exception of the component from iWorx, were generated using a mill and lathe. The base and the screws used to shift the platform on the *x*-axis and those used to tighten the electrode holder are made of homopolymer acetal (Delrin). The two electrode holders are made of chemical-resistant polyphenylene sulphide (PPS) plastic rods. A piece of laboratory tape is placed on top of the electrode block to hold the two wired electrodes securely in place. The entire platform is placed inside a Faraday cage created in-house and onto three vibration-absorbing mats (B004LYGH3U; Isolate It!) to minimize the number of mechanically induced artifacts in the trace.

### Zebrafish anesthetic

To generate the anesthetic solution, a tricaine (A5040-25G; MilliporeSigma) stock solution was first prepared: 15.31 mmol/l buffered to pH 7-7.5. The working solution was prepared by using 4.2 ml of the tricaine stock and adding up to 100 ml of system water from the zebrafish facility for a final concentration of 0.643 mmol/l. When not in use, stock tricaine was stored at 4°C. Fresh tricaine working solution was prepared before each ECG recording session.

### ECG recording protocol and materials

Details of the protocol are outlined in the Results section. This section provides additional details and materials not already reported.

A magnifying glass lamp (LTS-F21-61; Intertek) was attached to the table on which the ECG recording occurred to allow the operator to have a better view in order to insert the electrodes into the zebrafish. Electrodes were loosely taped to the recording table using standard laboratory tape to prevent entanglement during protocol operation. The piece of sponge into which the ground electrode was inserted had the following dimensions: 3.175×0.635×0.635 cm.

All ECG signals were digitized using the PowerLab 8/30 (ML70; ADInstruments) and amplified with the Animal BioAmp (ML136; ADInstruments). ECGs were recorded using LabChart version 7.3.8 (ADInstruments) for Windows, with a LabChart Pro license.

Data for this manuscript were derived from a total of 245 traces collected from 205 unique fish. Traces were collected from fish across different experimental conditions (drug or vehicle-control dosing experiments and non-dosing experiments) and different recording locations, from fish of varying genotypes (AB wild-type, wild-type from both the *kcnh6a^s290^* and *kcnh6a^tb218^* mutant lines, *kcnh6a*^s290/+^) and from fish of both sexes (102 females and 103 males). Each fish was assigned an identification number that was continuous through a specific genotype or drug condition to allow for tracking (i.e. AB-11 and FA-11 are two different fish). In addition to body size parameters and trace abnormalities, the following information was also noted in the recording manifest during the recording session: date of analysis, date of birth, age, genotype, stock number, mortality and ECG recording location.

To apply digital filters in LabChart, multiple channels must be generated to layer the digital filters on top of the ‘raw’ ECG signal with only the hardware filters applied. Therefore, our LabChart interface used three separate channels to generate the filtered trace used for downstream analyses. Channel 1 was the ‘raw’ ECG signal, with only the hardware filters applied. Channel 2 was where the low-pass filter was applied; the source channel was selected as Channel 1, and the transition width was set to be auto-adjusted. Channel 4 was where the high-pass filter was applied; the source channel was selected as Channel 2, and the transition width was set to be auto-adjusted. The ECG signal from Channel 4 was the final filtered trace that we used for all downstream analyses.

### LabChart ECG trace analysis

All LabChart (https://www.adinstruments.com/products/labchart) analyses were completed in LabChart version 7.3.8 using version 2.3.2 of the ‘ECG Analysis’ add-on. The last 120 s in the portion of the 180 s recording captured after the adjustment period ended was first selected for analysis. The following options were then selected within the settings for the ‘ECG Analysis’ add-on that was required for trace analysis: ‘Pig’ preset for Detection and Analysis settings, Block averaging for Averaging, Bazett for QTc, and QRS maximum for Alignment. All beats identified using the above settings were classified as ‘Good’ and therefore included in the calculation of the average trace. QRS complex identification was then checked manually by the analyst to ensure that all QRS complexes were marked correctly by LabChart. The analyst then selected ‘ECG Averaging View’ and manually identified the P start, P peak, P end, QRS start, QRS end, ST height, T peak and T end within the generated average trace by moving the appropriate lines. After all markers were placed correctly, the analyst navigated to ‘ECG Table View’ in order to export a .txt containing the measurements that were used for downstream statistical analyses. All columns available within the ‘ECG Table View’ were selected to be displayed in the .txt file; in addition, intervals were selected to be shown in milliseconds to four significant figures, and summary information was also selected for inclusion.

### ECG trace quality control

After each ECG recording session, an analyst manually examined every ECG trace through the LabChart interface to assign a quality score to the trace. The grading was based only on the segment of the recording to be used for downstream analyses (i.e. none of the recording captured during the adjustment period was examined). The analyst was not blinded regarding the experiment from which the traces to be graded derived (i.e. if the traces came from an FA-dosing experiment) but they were blinded regarding the experimental group the to which fish belonged (i.e. if the fish was part of the control or FA-dosed group). Each ECG trace was assigned a quality score ranging from 1 to 5, with an additional score of 7 used to describe traces in which the recording was halted owing to the inability to obtain any signal resembling an ECG, even after adjustment. Details regarding the grading criteria for scores 1-5 can be found in Fig. S3. In addition to assigning the quality score, the analysts noted whether the trace contained cycles (one P wave, one QRS) in which the P wave amplitude exceeded the R wave amplitude and whether the trace contained any arrhythmias or other abnormalities. Both the quality score and the abnormalities were then noted in the manifest file. In addition, after the trace was analyzed using the ‘ECG Analysis’ module, the analyst examined the ‘Average Trace’ plot for each ECG recording to ensure that the annotation regarding the P wave and R wave amplitudes was appropriate. According to our established pipeline, we move forward only with heart rate, interval and amplitude calculations with traces that have a quality score of 1-4.

### Calculation of ECG protocol procedure time

We obtained the start and end timestamps (e.g. 12:43) for all traces collected through our recording protocol by using the LabChart interface. The start time was considered as the timestamp when the adjustment period began. The end time was considered the timestamp when the entire recording was stopped and not only the timestamp when the 120 s segment used for analysis ended. To obtain these timestamps, the ‘Display Settings’ of each recording was changed such that Time of day was selected in the ‘Time Format’ box. The protocol procedure time for the *j*^th^+1 recording on day *y* as part of session *x* was calculated as the time difference between the end time of the *j*^th^+1 recording and the end time of the *j*^th^ recording also captured on day *y* as part of session *x* using difftime(). By nature of this calculation, the first and last recording collected for a particular session was excluded. We also excluded the first recording that was captured immediately after a substantial break occurred within the session; by default, the second recording occurring after the break was also excluded. Additionally, recordings graded with a QC score of 7 were not included, nor were recordings from experiments that did not require fish to be dosed in drug or vehicle control, because the time-sensitive nature of those experiments would inherently increase protocol time. After exclusions, we were left with 179 traces from a total of 143 unique fish that were captured across different experimental conditions using fish of varying genotypes (AB wild-type, wild-type from both the *kcnh6a^s290^* and *kcnh6a^tb218^* mutant lines, *kcnh6a^s290^*^/+^) and of both sexes (82 females and 61 males).

### zERG development, ECG trace analysis and calculation of ECG measurements

Initial creation and development of zERG was completed using MATLAB (https://www.mathworks.com/products/matlab.html) version 9.4 and GUIDE version 2.5 with the following dependent add-ons: Signal Processing Toolbox version 8.0 and Image Processing Toolbox version 10.2. Throughout the development process, zERG was run on MATLAB versions 9.6 and 9.7 and GUIDE version 2.5 with the following dependent add-ons: Signal Processing Toolbox version 8.3 and Image Processing Toolbox version 11.0. Final zERG scripts used for all analyses in the manuscript used MATLAB version 9.7 and GUIDE version 2.5 with the following dependent add-ons: Signal Processing Toolbox version 8.3 and Image Processing Toolbox version 11.0.

For each recording analyzed in zERG, the following method was used. After choosing to ‘Run’ the script in MATLAB, the working directory containing all .mat and/or .txt to be analyzed was selected. Once the working directory was selected, the zERG user interface appeared (Fig. S6), with all .mat and .txt within the selected working directory listed within the ‘Data Files’ box. A single ECG recording was selected for analysis (highlighted in gray within the ‘Data Files’ box), and the entire recording was plotted within the ‘Full Recording’ section of the GUI. ‘Use Full Recording’ was then checked because the .mat file exported from LabChart was already trimmed to the appropriate time frame for analysis. After selection of the entire trace, the standard zERG analysis pipeline was used.

First, ‘Start Peak Analysis’ was selected, triggering the entire trace to be plotted in the ‘Peak Analysis’ box. Initial peak identification was completed as described in the Results section using the findpeaks function, requiring a minimum peak height threshold, for which all maxima above this user-defined threshold are called a peak, and an initial user-defined minimum peak interval. The result of this first step was the identification of all P waves (labeled with a red asterisk) and QRS complexes (labeled with a black asterisk) within the plot in the ‘Peak Analysis’ box.

The method to edit the initially identified P waves and QRS complexes was subjective and depended on the number and level of trace artifacts in the ECG recording. No uniform editing path was used, but, in general, individual P waves and QRS complexes were added and/or deleted by selecting (via the ‘Select Peak’, ‘Box Peaks’ or ‘Deselect’ buttons) the ‘Add P’, ‘Add QRS’, ‘Swap Colors’ or ‘Delete Peaks’ buttons. When the trace contained a high level of mechanically induced artifacts and extraneous features in the trace had been erroneously marked as P waves and/or QRS complexes, the ‘Noise Remover’ function was used to automate wave marker identification and correctly identify the P waves and/or QRS complexes using a modified version of the findpeaks function. For traces with abnormal QRS morphology, the minima of each ECG cycle were found instead. This second step resulted in a trace within the ‘Peak Analysis’ box where all P waves and QRS complexes were correctly identified and labeled or where all minima were correctly identified and labeled.

After completing the ‘Peak Analysis’ portion of the GUI, ‘Calculate Average Trace’ was selected. This step first determined a plotting window that was applied to each cycle within the trace to determine how much of each cycle was plotted (i.e. how much of the trace before the P wave and after the QRS complex should be considered as one ECG cycle). After identification of the plotting window, each ECG cycle in the trace was plotted and aligned by the R wave; this resulted in a compiled trace of every cycle within the recording. If minima were identified in the ‘Peak Analysis’ step, the average trace was aligned via the minima. In both cases, an average trace was calculated by taking the mean of all voltage measurements at each point within each ECG cycle. An isoelectric line was also plotted, calculated as the median of all points before the Q wave. For each point in each cycle, the *y*-coordinate was a voltage measurement, and the *x*-coordinate was the position of that point in relation to the ECG cycle. Therefore, the first data point in the ECG cycle had an *x*-coordinate of one. All *n* points were plotted, with the last data point in the ECG cycle being placed at the *x*-coordinate of *n*.

Next, ‘Add Markers’ was selected to begin the identification of the following features on the average trace: (1) P wave start, (2) P wave peak, (3) P wave end, (4) Q wave, (5) R wave, (6) S wave, (7) T wave start, (8) T wave peak and (9) T wave end. For each of these markers, a line appeared on the average trace. Each line was then moved to the appropriate position on the average trace, along the *x*-axis; the position of the line along the *x*-axis was saved to be used for measurement calculation. Once the lines were placed correctly, ‘Confirm Markers’ was selected. This last step performed the calculation of measurements that appear in the results .txt.

The number of cycles (nTraces) was calculated as the length of the matrix that contains the coordinates of each R wave in the recording. The RR interval and heart rate were calculated using the coordinates in the ‘Peak Analysis’ section. For every *j*^th^ R wave, the RR interval for the *j*^th^ cycle was calculated as the *x*-coordinate of the *j*^th^+1 R wave minus the *x*-coordinate of the *j*^th^ R wave. Once this loop was finished for the entire recording, the mean RR interval (in ms) was calculated; heart rate (in bpm) was then calculated as 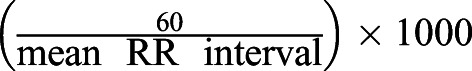. For the remaining measurements, the calculations were based solely on the positions of the markers on the average trace. For this reason, zERG first sorted the *x*-coordinates of the nine lines in order from smallest to largest; the smallest *x*-coordinate was considered as the position of line 1, and the largest *x*-coordinate as the position of line 9. After this sorting, the intervals were calculated by subtracting the *x*-coordinates of the corresponding lines. The PR interval was calculated as the *x*-coordinate of line 4 minus line 1. The QRS interval was calculated as the *x*-coordinate of line 6 minus line 4. The QT interval was calculated as the *x*-coordinate of line 9 minus line 4. Wave amplitudes were calculated as the distance from the peak of each wave to the point on the isoelectric line that was directly perpendicular to the peak.

With the exception of traces collected from the AB multiple timepoint ECG recording, all heart rate and interval measurements used for this manuscript used algorithms in zERG version 1.0. Traces from the AB multiple timepoint experiment were analyzed using zERG version 1.1. There were no differences in the algorithms used to calculate heart rate and ECG intervals between versions 1.0 and 1.1. However, differences exist in the amplitude calculations and the wave detection methods; we recommend that analysts use the latest version of zERG to examine their data.

Additional details regarding zERG, the different versions available and the calculations performed by the program can be found on the GitHub repository.

### Conversion and export of LabChart ECG traces into MATLAB file format

To export the .mat file of the LabChart data for zERG analysis, the last 120 s in the portion of the 180 s recording captured after the adjustment period ended was highlighted. The following options were then selected after choosing ‘Export…’ from the ‘File’ menu: ‘Data’ and ‘32-bit floating point’ within the ‘Include’ box and ‘Upsample to same rate’ within the ‘Sampling rate’ box. Only the channel containing the filtered trace (Channel 4) using the previously described hardware and digital filters was exported into the .mat file for zERG analysis.

### Tracking zERG analysis time

zERG analysis time was tracked using zERG version 1.0. The start time was considered as the point when the analyst selected the trace to be analyzed. The end time was considered as the point when the analyst selected ‘Analyze ECGs’ and measurements were obtained. The analyst was not blinded with respect to the experiment from which the traces derived (i.e. whether the traces came from an FA-dosing experiment) but they were blinded with respect to the experimental group to which the fish belonged (i.e. whether the fish was part of the control or FA-dosed group). We excluded traces with quality scores of 5 and 7, in addition to traces with abnormal waveforms or substantial noise that prevented zERG from accurately identifying P waves and QRS complexes and/or generating an accurate average trace. After exclusions, we examined the analysis time for 182 traces collected from 151 unique fish across different experimental conditions (drug or vehicle-control dosing experiments and non-dosing experiments), fish of varying genotypes (AB wild-type, wild-type from both the *kcnh6a^s290^* and *kcnh6a^tb218^* mutant lines, *kcnh6a*^s290/+^) and sex (78 females and 73 males).

### zERG and LabChart comparison, manual heart rate calculations

We used traces recorded from 11 AB wild-type fish (eight females and three males; total of 41 traces) at multiple timepoints, in addition to traces recorded from drug studies involving 0.800 mmol/l FA (ten from the control group: two females and eight males; four from the FA group: three females and one male; total of 14 traces). All traces used for this analysis passed our quality score criteria. In total, we examined measurements from 55 traces for which both zERG and the ‘ECG Analysis’ module in LabChart were able to identify ECG peaks correctly; that is, we used only traces in which the P wave amplitude did not exceed the R wave amplitude. For the FA set, we did not include any traces with conduction blocks because LabChart might identify the consecutive P waves inaccurately as QRS complexes. Measurements were obtained using the LabChart and zERG trace analysis protocols as described in earlier sections. Experimental conditions for the AB multiple timepoint and 0.800 mmol/1 FA are described in the Results section and in later sections within the Materials and Methods.

Manual heart rate calculations were completed for only the 14 FA traces. For each of these traces, the number of R waves occurring within the 120 s selected for data analysis was counted. The number was verified against two plus the number of ‘Used’ beats obtained through .txt output from the ‘ECG Analysis’ module in LabChart; the adjustment was made to include the first and last beat, which LabChart excludes from the ‘ECG Analysis’ module. The total number of manually counted R waves was then divided by the total recording time (in min) to obtain the manual rate (in bpm).

### AB multiple timepoint ECG recordings

Traces were captured from a total of 15 AB wild-type fish ∼6 months of age over four consecutive days. On day 1, each fish was assigned an identification number. On days 2-4, the identification number was randomized to dictate recording order. Before analysis, in addition to excluding traces based on poor quality score, we excluded traces from three fish that died during the 4 day time frame, bringing the total number of individual fish and traces used for our analysis to 11 (eight females and three males) and 42, respectively. To ensure that we recorded the same fish over the 4 days and prevent misidentification, each fish was housed temporarily in a plastic cup for the duration of this experiment; fish were returned to their tanks at the end of day 4. During recording and zERG analysis, analysts were not blinded with respect to which identification number belonged to each fish or which day each trace was collected, in order to maintain proper trace assignment and recall.

### ECG recordings of fish used for correlation analyses, regression model development, average trait values and power calculations

This dataset was derived from the combination of recordings captured from AB wild-type fish collected across six different recording sessions, AB wild-type fish as part of the AB multiple timepoint recordings and wild-type fish from two different lines: *kcnh6a^s290^* and *kcnh6a^tb218^*. All fish were in the age range of 3-6 months. Experimental protocols for fish used for the multiple timepoint recordings were conducted as described in earlier sections; only measurements from day 1 were included in this dataset. For recording sessions in which *kcnh6a^s290^* and *kcnh6a^tb218^* traces were captured, given that other genotypes were also recorded, analysts were blinded to the genotypes of each fish; in addition, the recording order among the different genotypes was randomized. In total, ECGs used for this analysis were collected from fish across 13 different recording sessions and two different recording locations. All traces were collected using the standard recording protocol.

All traces passed the quality score criteria. During zERG analysis, the analyst was not blinded with respect to the recording session from which the traces came; they were also not blinded to fish genotype and fish line (AB, *kcnh6a^s290^* or *kcnh6a^tb218^*). The initial dataset after filtering based on quality score consisted of 79 traces. After excluding one trace with abnormal waveforms that prevented zERG version 1.0 from accurately identifying P waves and QRS complexes, and generating an accurate average trace and removing eight outliers by visual inspection of the distributions of heart rate and the intervals, 70 traces (one trace per fish) were used for these two different analyses. The final distribution of genotypes and sexes were as follows: AB, 32 females and 15 males; *kcnh6a^s290^*, eight females and 12 males; *kcnh6a^tb218^*, two females and one male.

### FA stock solution

FA (F6777; Sigma-Aldrich) was dissolved in DMSO (472301-100ML; MilliporeSigma) to make an 84.3 mmol/l stock solution. When not in use, the FA stock solution was stored in a darkened glass vial at 4°C.

### FA and DMSO dose-response observations

The 84.3 mmol/l stock solution was used to generate 5 ml FA at each of the various concentrations outlined in Table S3. The various DMSO solutions were generated by dissolving the appropriate amount of DMSO in deionized water. For each FA or DMSO concentration, one AB wild-type fish was placed in a glass beaker containing the drug or vehicle control and was observed every 10 min for 1 h by the same analyst, who was blinded with respect to dosing conditions. Behaviors such as swim depth (whether the fish was swimming at the top, middle or bottom of the glass beaker), swim rate, lack of movement and erratic movement were observed. Additionally, survival after the hour of dosing was noted. Experiments were conducted over a span of 2 days. Fresh FA dosing solution was made before each day.

### Treatment with 0.800 mmol/l FA and ECG recordings

To generate the 0.800 mmol/l FA dosing solution, 2.85 ml of the 84.3 mmol/l FA stock solution was added to 297.15 ml system water. The combined FA and tricaine solution was created by adding 2.85 ml of the FA stock and 12.6 ml of 0.643 mmol/l tricaine to 284.55 ml system water. The control 0.95% DMSO dosing and anesthetic solutions were made using the same volumes as described above but substituting DMSO for FA. Each dosing solution was then divided evenly into two glass beakers to allow for an efficient recording schedule, where up to two fish could be dosed at the same time at different time intervals (i.e. the dosing of the second fish begins when approximately one-third of the dosing time for the first fish has passed). Fresh FA dosing solution was made before each independent experiment.

The ECG recording protocol used was as described earlier, with the following modifications. Each fish was assigned an identification number and then placed into a glass beaker containing either 0.800 mmol/l or 0.95% DMSO solution for the specified time; this dosing occurred within the zebrafish housing facility to account for potential effects of ambient room temperature. The fish was then anesthetized with the corresponding combined anesthetic solution. Upon sufficient anesthetization, the fish was brought out of the housing facility and subjected to the standard ECG recording protocol. Operators were blinded with respect to which treatment each fish received, and the recording order of fish from each treatment was randomized.

Initial sample sizes for the FA experiments were as follows: 20 (for the experiment using 5.2-month-old fish) and 30 (for the experiment using 3.5-month-old fish). After excluding traces based on quality score and traces that were not able to be analyzed by zERG version 1.0, final samples sizes were 15 (DMSO vehicle-control *n*=10, four females and six males; FA *n*=5, three females and two males) and 21 (DMSO vehicle-control *n*=10, two females and eight males; FA *n*=11, five females and six males), for the respective experiments. During zERG analysis, analysts were also blinded with respect to which treatment each fish received. Each specific experiment was completed once within the laboratory.

### *kcnh6a^s290^* ECG recordings

ECG recordings for *kcnh6a^s290^* occurred over five separate sessions across two different locations. In each session, analysts were blinded to fish genotype. The order of recording was randomized among the fish examined in each recording session. After excluding poor quality traces, traces with abnormal waveforms that prevented zERG version 1.0 from accurately identifying P waves and QRS complexes and generating an accurate average trace, and after removing outliers by visual inspection of the distributions of heart rate and the intervals, the final set for group comparison included 54 fish. The genotype and sex distribution were as follows: wild-type, *n*=22 with nine females and 13 males; heterozygous, *n*=32 with 16 females and 16 males.

### ECG image trace acquisition

The trace from the adult male (record ID: 16265) used in Fig. 1 was obtained from the publicly available MIT-BIH Normal Sinus Rhythm Database (Goldberger et al., 2000). Voltage measurements and corresponding times were downloaded using the PhysioBank ATM under the following options: Signals, All; Annotations, reference beat and signal quality annotations (atr); Length, 1 min; Time format, seconds; Data format, standard; Toolbox, Show samples as text. The voltages were then imported into LabChart, and an average trace was generated using the Human preset in the ‘ECG Analysis’ module.

Zebrafish ECG traces used for figures were captured through LabChart, by using either the ‘Zoom View’ or the ‘Average View’ available as part of the ‘ECG Analysis’ module.

### Statistical analysis

All graphs and analyses were completed in R version 3.6.3 (https://www.r-project.org/) using R Studio version 1.3.1073 (https://www.rstudio.com/products/rstudio/). Custom R scripts were written and used for all statistical analyses. Linear regression was completed using lm() and included covariates as described. All mixed effect models were generated using lmer() within the lme4 package version 1.1-23 and included fixed and random covariates as described. For all box plots, the boxes represent the upper and lower quartiles and the whiskers represent variability; the median is illustrated as the black line within the box. Differences were considered significant at *P*<0.05. Additional details not already reported in the Results section are provided below.

#### AB multiple timepoint

A linear mixed model was used to obtain the variances explained by the between- and within-fish terms. The model was defined as: ECG Trait∼(1|fish identification variable)+(1|date of trace collection), where the first random effect is the between-fish term and the second is the within-fish term. The total variance explained was calculated by taking the sum of the variance explained by the between-fish term and the within-fish term, in addition to those of the residuals. The percentage of the total variance explained by each term was then calculated as 100 multiplied by the variance explained by the term divided by the total variance. The ratio was then calculated as the ratio of the percentage of the total variance explained by the between-fish term to the percentage of the total variance explained by the within-fish term. Variance-explained values <5×10^−4^ were rounded to zero before calculating the percentages.

To generate the null model in which there are no differences in the variance explained by the between- and within-fish terms, we permuted the fish identification variable such that the 42 traces were randomly assigned to different fish across the 4 days of recording. We then repeated the above analysis to obtain the percentage of the total variance explained for the between-fish and within-fish terms, in addition to the ratio.

#### zERG and LabChart comparison

The *R*^2^ values were determined using a linear regression between the ECG metric calculated by zERG and the corresponding metric calculated using the ‘ECG Analysis’ module in LabChart.

#### Regression model development

After residuals were obtained from a linear mixed model adjusting the ECG trait for age, recording location and date of trace collection, model selection based on AIC values using a forward regression approach was completed using step()*.*

#### FA drug effect

For each independent experiment, traces were recorded in one recording session, fish were all the same age and the recordings occurred in the same location. Therefore, we did not adjust for date of ECG recording, age or recording location. The *P*-values were obtained from a linear regression adjusting for sex and weight; the QT interval was additionally adjusted for heart rate.

#### *kcnh6a^s290^* phenotyping

To account for differences in recording sessions conducted with fish from the *kcnh6a^s290^* line, we used a linear mixed model to analyze measurements obtained from the traces, with recording date added as a random effect and with age and location of ECG recording added as fixed effects. Additional covariates included sex, weight and heart rate as determined through model selection and correlation analyses for each respective ECG trait.

#### Power calculations

A linear mixed model was first used to adjust measurements of all ECG traits and obtain residuals; covariates included recording date (as a random effect), age of fish and location of ECG recording as fixed effects. Additional fixed effect covariates included sex, weight and heart rate (for QT interval only) as determined through model selection and correlation analyses for each respective ECG trait. Power calculations were then performed using power.t.test() after specifying the standard deviations of the residuals for each trait, the power, type (‘two.sample’), alternative (‘two.sided’) and delta (defined as the difference in means such that the percentage difference in means will be equivalent to the percentage defined in Table 2).

## Supplementary Material

Supplementary information
