# Supplementary material for: Development and optimization of an in vivo electrocardiogram recording method and analysis program for adult zebrafish
Source: Dis Model Mech. 2021 Aug 11;14(8):dmm048827. doi: 10.1242/dmm.048827 (PMC8380046; doi:10.1242/dmm.048827)
Supplement: Supplementary information [file dmm-14-048827-s1.pdf]

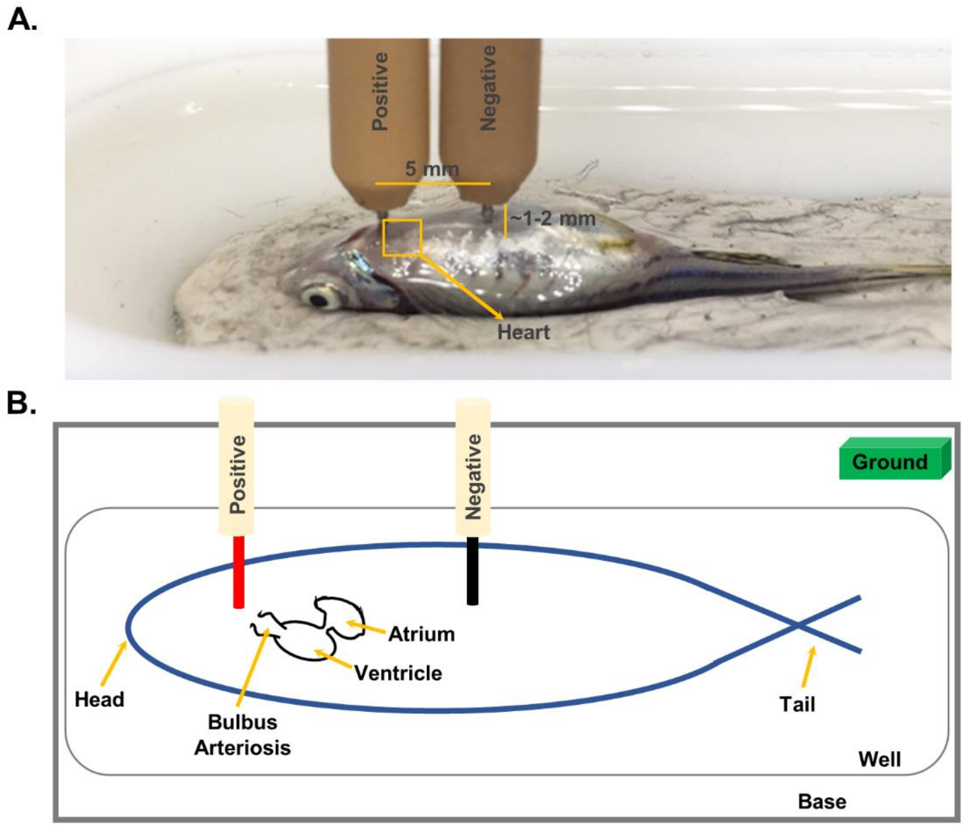

Fig. S1: Electrode Placement in Relation to Adult Zebrafish Body. (A) Photograph of an adult female AB wild-type zebrafish approximately 5 months of age on the ECG recording apparatus. Positive and negative electrodes are inserted into the normal positions required for recordings. (B) Illustration of positive, negative, and ground electrode placements, with respect to the zebrafish heart. Objects and positions are not necessarily drawn to scale but are relative to one another; the heart has been enlarged for viewing purposes.

Disease Models & Mechanisms • Supplementary information

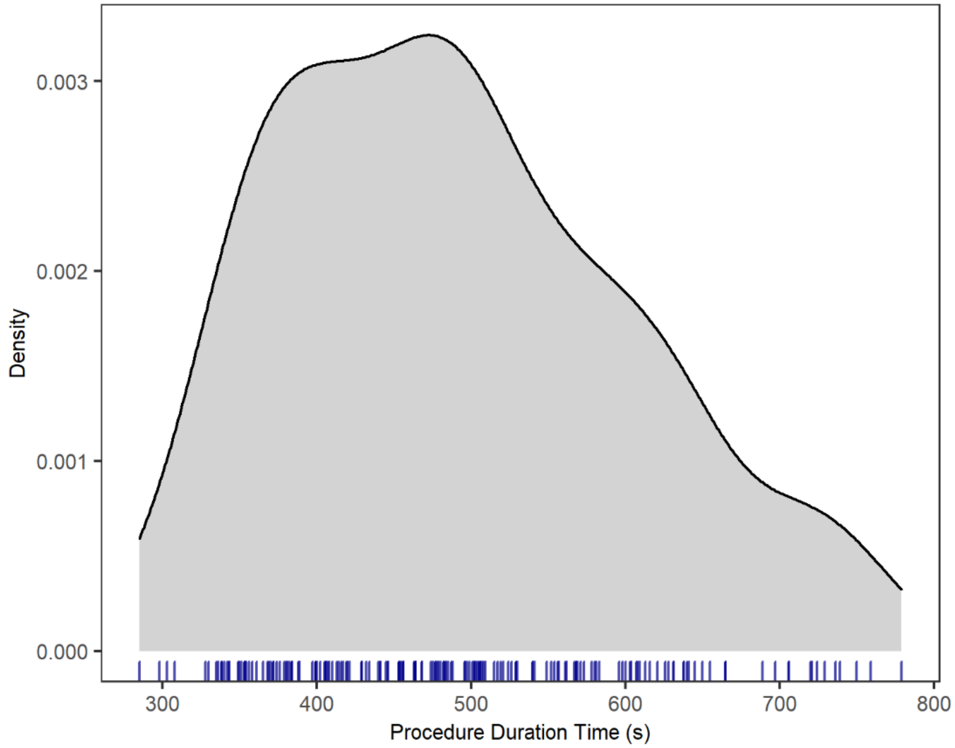

Fig. S2: Adult zebrafish ECG protocol procedure time. Rug plot illustrating the distribution of the adult zebrafish ECG protocol procedure time for 179 traces obtained using the described apparatus and protocol.

Disease Models & Mechanisms • Supplementary information

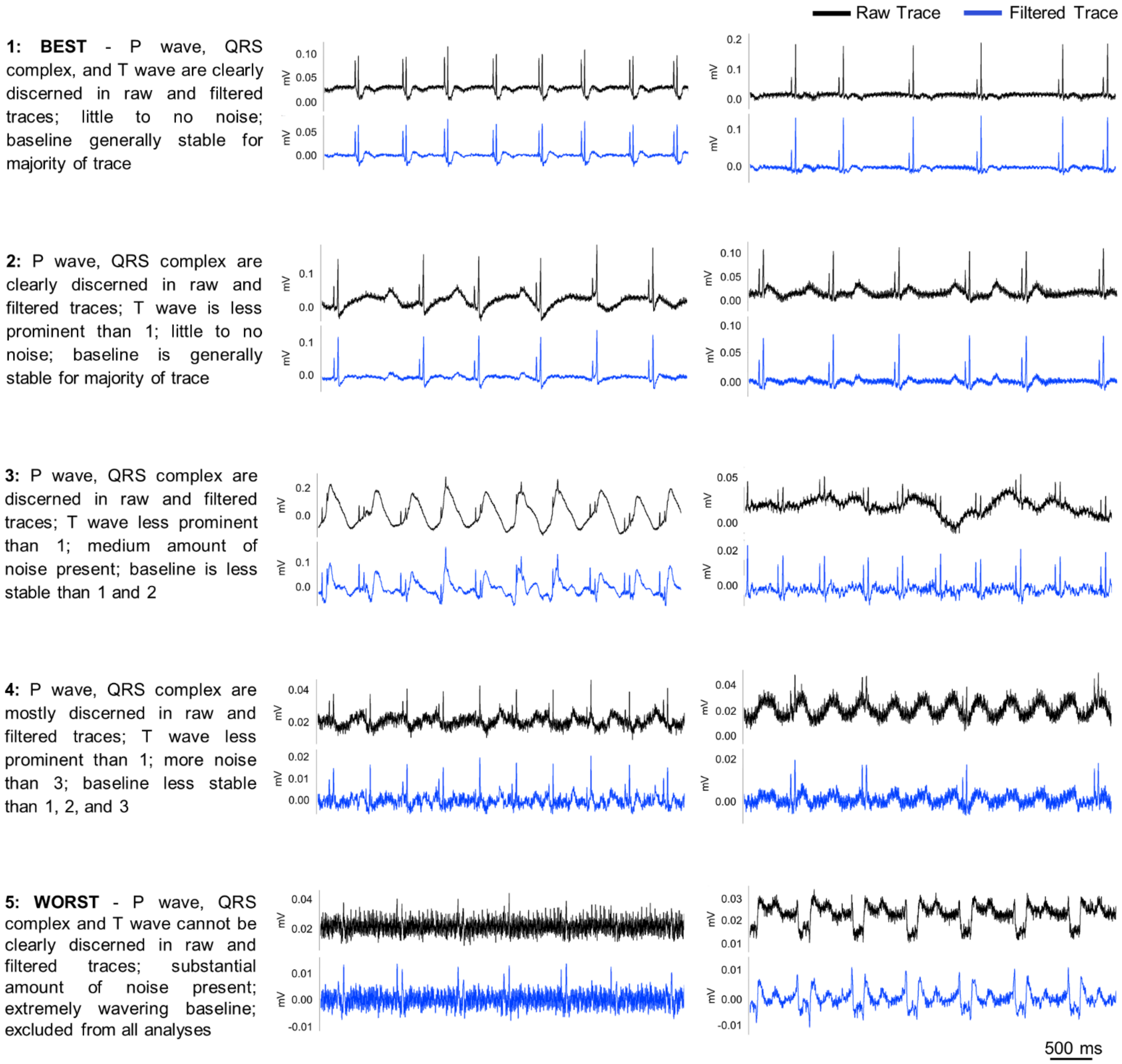

Fig. S3: ECG quality score criteria. Representative ECG traces with a specific quality score, ranging from 1-5. The left-most column contains a short description of the features that traces must meet in order to be assigned a particular score. Both the ‘raw’ (black, from a channel where only hardware filters are applied) and the ‘filtered’ (blue, from a channel where additional digitals filters are applied) were considered when scoring ECG traces. Only the ‘filtered’ trace from each recording was used for all downstream analyses. An additional quality score of 7 was assigned to traces where the recording was halted due to the inability to obtain any signal resembling an ECG,

Disease Models & Mechanisms • Supplementary information

even after adjustment. For each score, representative traces recorded from two different traces are shown. Traces were recorded from 9 unique AB wild-type fish (5 females, 4 males) as part of different experimental conditions (drug or vehicle-control dosing experiments and non-dosing experiments). Trace images were captured using Labchart ‘Zoom View.’

Disease Models & Mechanisms • Supplementary information

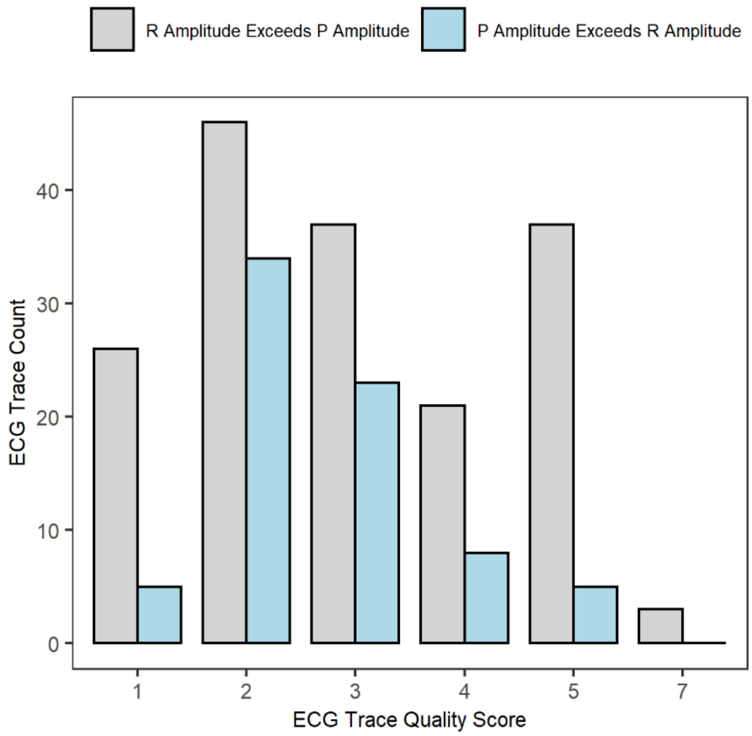

Fig. S4: Distribution of ECG Traces by quality score. Histogram showing the count of traces with a specific quality score. A total of 245 traces collected from 205 unique fish were tabulated.

Disease Models & Mechanisms • Supplementary information

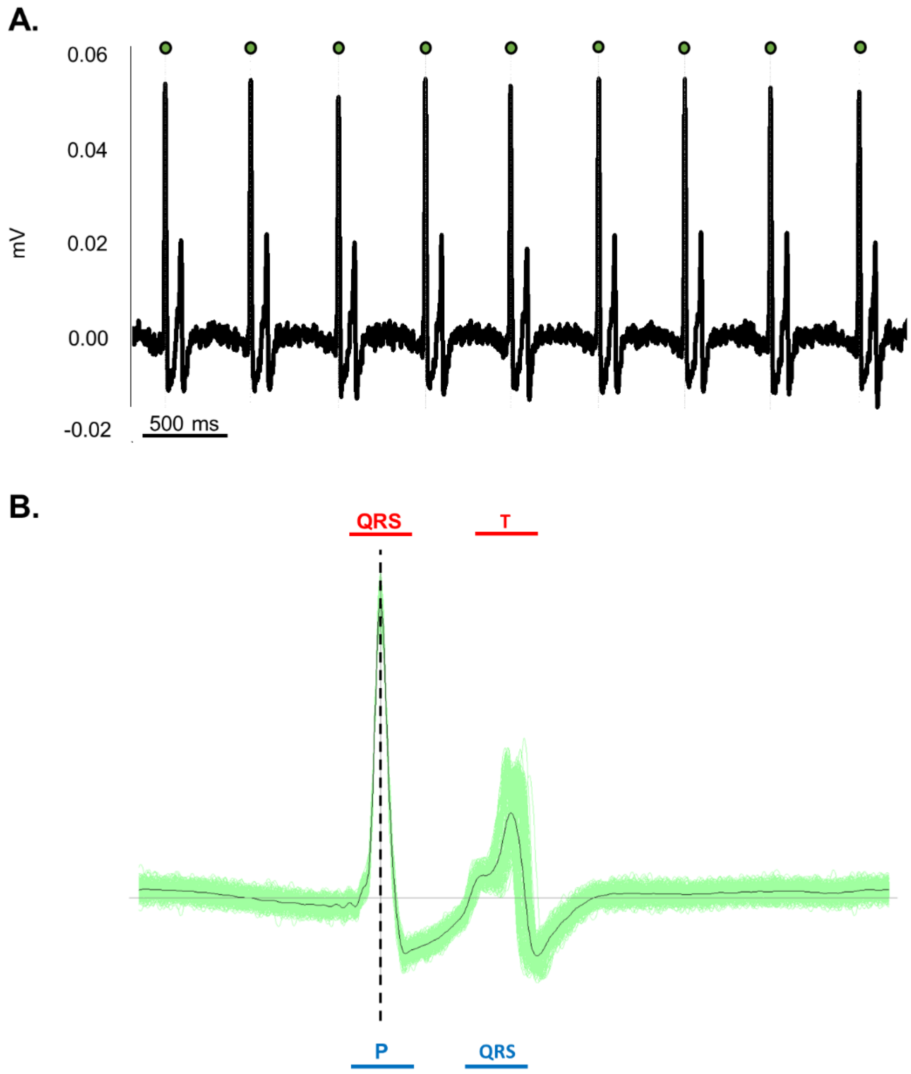

Fig. S5: ECG wave misidentification by Labchart, with traces where the P wave amplitude exceeds the R wave amplitude. (A) Representative ECG trace as viewed in Labchart; the green dots indicate the wave that the ECG Analysis module designated to be the QRS complex. The trace was collected from a 5.2-month-old female fish dosed with 0.800 mmol l^{-1} FA for 15 minutes. (B) The compiled average trace within Labchart of the entire 120 s recording of the fish shown in (A); the red text shows the calling of the average trace, due to the incorrect placement of the QRS maximum (black dashed line). The blue text indicates the correct calling.

Disease Models & Mechanisms • Supplementary information

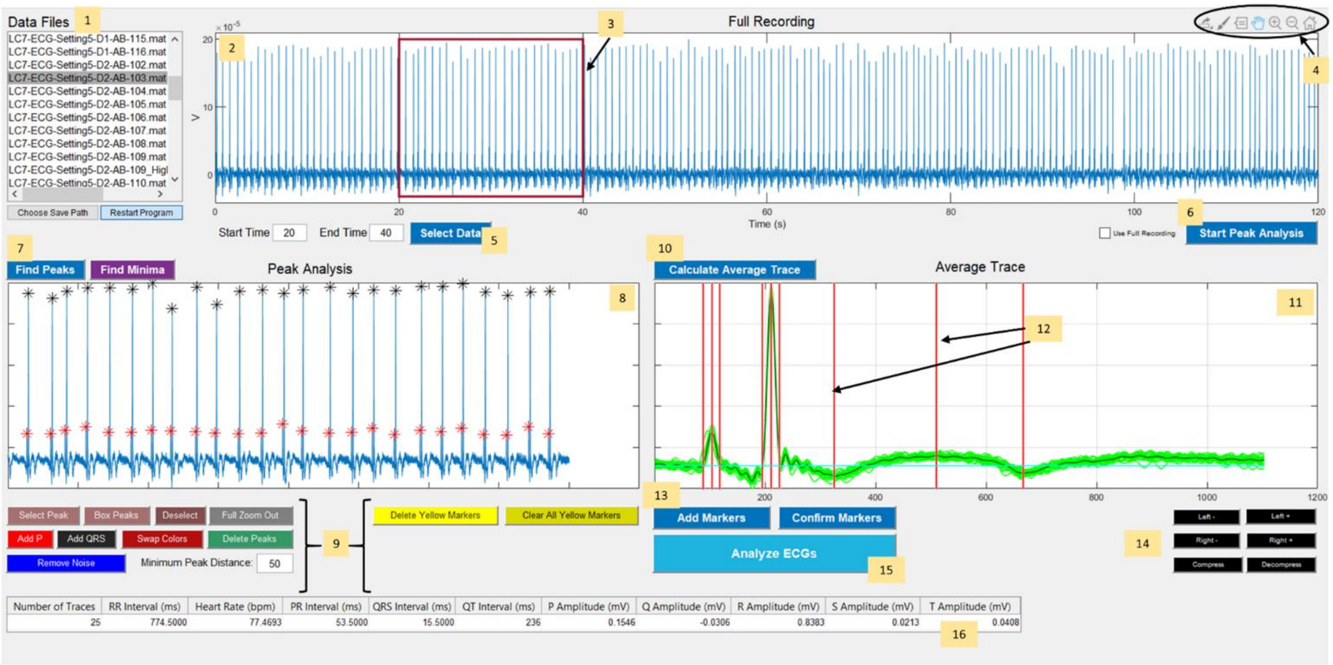

1: *Data Files* - List of .mat or .txt files in folder
2: *Full Recording* - Plot of trace (highlighted gray under *Data Files*)
3: *Red Rectangle* - Selected portion of trace for *Peak Analysis*
4: *Matlab Tool Bar*
5: *Select Data* – Choose potion of trace for *Peak Analysis*
6: Begin *Peak Analysis* of selected data
7: *Find Peaks* - Identify P and QRS complexes
8: *Peak Analysis* – Plot of trace (P - *; QRS - *)
9: Edit peak identification initially done through *Peak Analysis*
10: *Calculate Average Trace* – Align peaks average trace
11: *Average Trace* plot of selected data
12: Wave markers after confirmation
13: *Add Markers* – add markers to *Average Trace*
14: Edit *Average Trace* window
15: *Analyze ECGs* – output measurements and plots
16: Table listing ECG measurements

Fig. S6: zERG user interface. Name and description of the functions and/or buttons that each number indicates are detailed in the bottom portion of the figure. The interface was taken from zERG version 1.1.

Disease Models & Mechanisms • Supplementary information

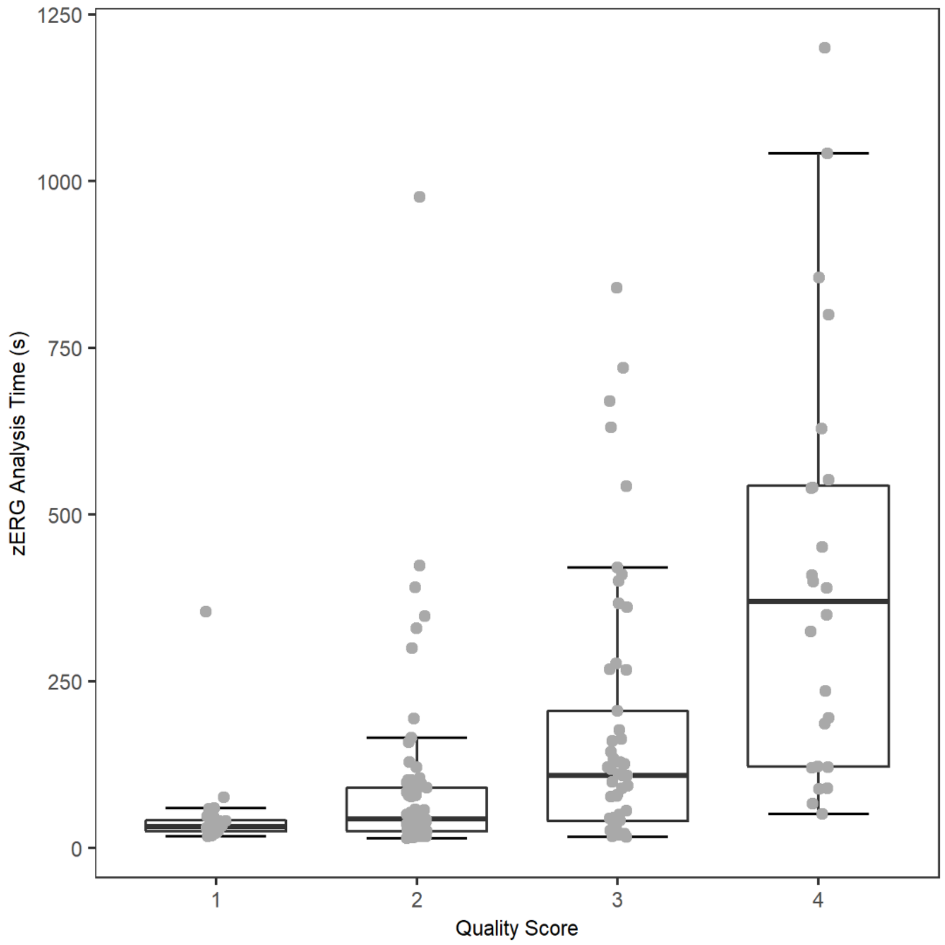

Fig. S7: zERG analysis time is highly dependent on ECG trace quality. Trace analysis time using zERG was tracked for a total of 182 traces.

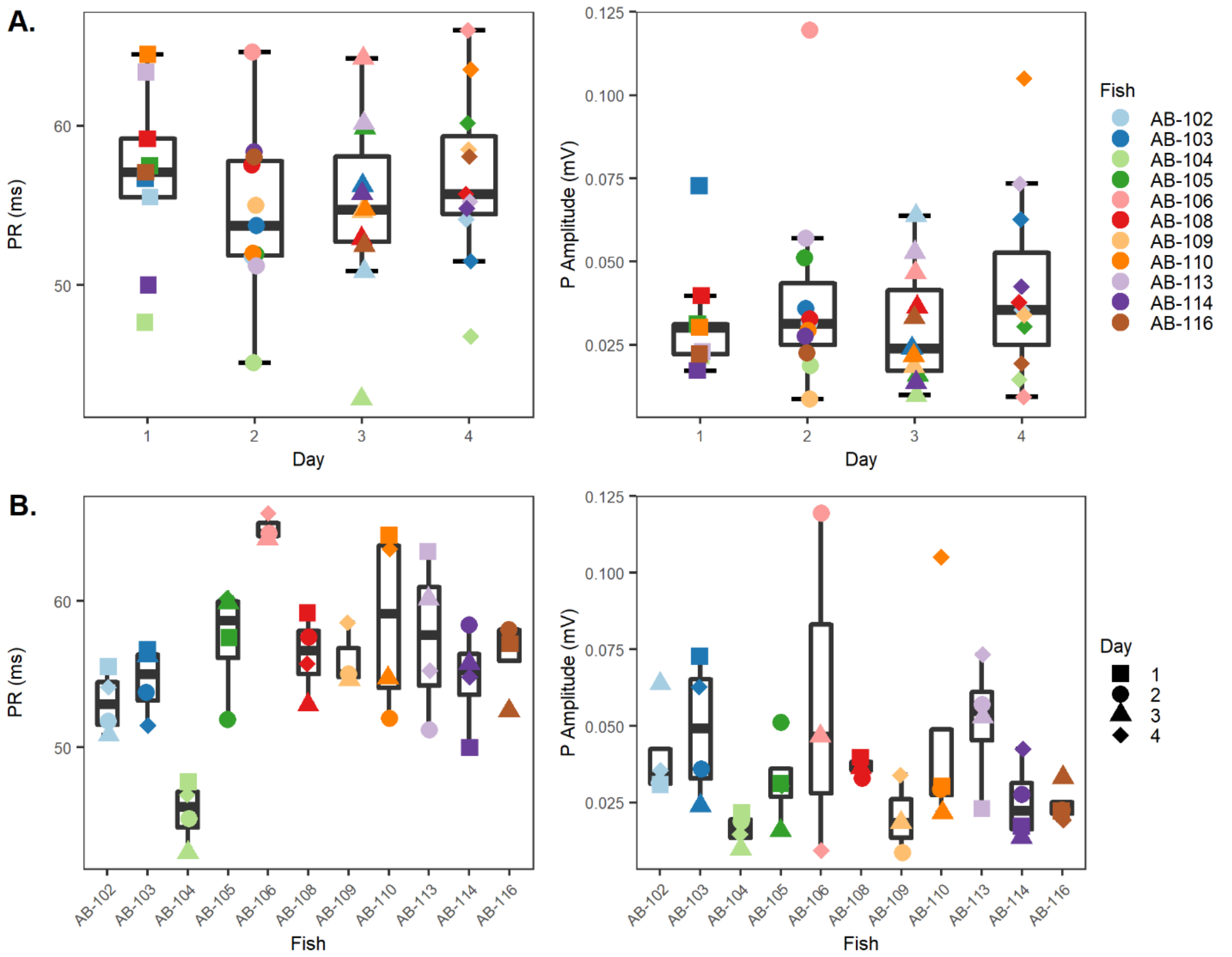

Fig. S8: Select measurements from AB wild-type fish captured over multiple timepoints. Over four consecutive days (Day 1,2,3,4) a total of 42 traces were captured from 11 different AB wild-type fish (fish identifier: AB-102 to AB-116) at 6 months of age using the described adult zebrafish ECG recording protocol. Interval and amplitude measurements were then calculated using zERG. Each color represents one of the 11 AB fish while the shape of the data point represents the day from which the measurement was taken. (A) PR interval (*left*) and P amplitude (*right)* of the 11 fish separated by day; these plots can be considered as the graphical representation of the between fish term. (B) PR interval (*left*) and P amplitude (*right)* of the 11 fish separated by fish; these plots can be considered as the graphical representation of the within fish term.

Disease Models & Mechanisms • Supplementary information

Table S1: Averages for adult zebrafish cardiac electrophysiology traits. Traces were recorded from a total of 70 wild-type fish approximately 3–6 months of age by combining both AB (including the first measurement of fish where we captured traces over multiple timepoints) and wild-type fish from two different lines: *kcnh6a*^{s290} and *kcnh6a*^{tb218}. Traces were captured during ECG recording sessions held on different days and across two separate recording locations. Averages in the second column are listed as (mean±s.d.). QT interval is uncorrected for heart rate.

**Trait**	
Heart rate (bpm)	101±17.3
**Interval (ms)**	
RR	615±115
PR	58.7±8.66
QRS	18.2±3.48
QT (Uncorrected)	191±49

Table S2: Adult zebrafish ECG trait model development. For each trait, term(s) that were included in the final model selected by the forward regression approach are listed. This analysis used measurements from traces captured from wild-type fish (3-6 months of age) by combining AB fish and wild-type fish from two different lines (*kcnh6a*^{s290} and *kcnh6a*^{tb218}) for a total of 70 fish.

**Trait**	**Covariates**
Heart rate	Sex
RR	Sex
PR	Sex
QRS	Sex, Weight
QT	Heart rate, Sex, Length, Weight

Table S3: FA dose-response observations. For each FA concentration, 1 adult (older than 3 months) AB wild-type fish was placed in a glass beaker containing the drug and observed every 10 minutes, up to one hour. Behavior such as swim depth (whether the fish was swimming at the top, middle, or bottom of the glass beaker), swim rate, lack of movement, erratic movement, and survival after the hour dosing was observed. Fish noted with * were approximately 1 year old at the time of the dosing.

Sex	Length (cm)	Weight (g)	Width (cm)	Dose (mmol l^{-1})	Time (min)	Swim Depth	Swim Rate	Static Position	Erratic Movement	Survival After 60 min
Female*	3.2	0.2714	0.6	0.001	0	Normal	Normal	No	No	
					10	Normal	Normal	No	No	
					20	Normal	Normal	No	No	
					30	Normal	Normal	No	No	
					40	Normal	Normal	No	No	
					50	Normal	Normal	No	No	
					60	Normal	Normal	No	No	Yes
Male*	3.1	0.2617	0.6	0.010	0	Normal	Normal	No	No	
					10	Normal	Normal	No	No	
					20	Normal	Normal	No	No	
					30	Normal	Normal	No	No	
					40	Normal	Normal	No	No	
					50	Normal	Normal	No	No	
					60	Normal	Normal	No	No	Yes
Male	2.9	0.2059	0.5	0.025	0	Normal	Normal	No	No	
					10	Normal	Normal	No	No	
					20	Normal	Normal	No	No	
					30	Normal	Normal	No	No	
					40	Normal	Normal	No	No	

					50	Normal	Normal	No	No	
					60	Normal	Normal	No	No	Yes
Male	2.9	0.197	0.5	0.050	0	Normal	Normal	No	No	
					10	Normal	Normal	No	No	
					20	Normal	Normal	No	No	
					30	Normal	Normal	No	No	
					40	Normal	Normal	No	No	
					50	Normal	Normal	No	No	
					60	Normal	Normal	No	No	Yes
Male	3.2	0.2476	0.6	0.075	0	Normal	Normal	No	No	
					10	Normal	Normal	No	No	
					20	Normal	Normal	No	No	
					30	Normal	Normal	No	No	
					40	Normal	Normal	No	No	
					50	Normal	Normal	No	No	
					60	Normal	Normal	No	No	Yes
Male	3.5	0.3162	0.6	0.100	0	Normal	Normal	No	No	
					10	Normal	Normal	No	No	
					20	Normal	Normal	No	No	
					30	Normal	Normal	No	No	
					40	Normal	Normal	No	No	
					50	Normal	Normal	No	No	
					60	Normal	Normal	No	No	Yes
Male	3.2	0.1782	0.5	0.250	0	Normal	Normal	No	No	
					10	Normal	Normal	No	No	
					20	Near bottom	Normal	Very little	No	
					30	Near bottom	Normal	Yes	No	
					40	Near bottom	Slow	Yes	No	
					50	Near bottom	Slow	Yes	No	

Disease Models & Mechanisms • Supplementary information

					60	Near bottom	Slow	Yes	No	Yes
Not Recorded*	3.3	0.2688	0.5	0.500	0	Low	Normal	No	No	
					10	Low	Normal	Yes	Occasionally	
					20	Low	Normal	Yes	Occasionally	
					30	Low	Normal	Yes	Occasionally	
					40	Low	Normal	Yes	Occasionally	
					50	Low	Normal	Yes	No	
					60	Low	Normal	Yes	No	No
Female*	3.9	0.3248	0.6	0.731	0	Low	Normal	No	No	
					10	Low	Normal	Yes	Yes	
					20	Low	Normal	Yes	Yes	
					30	Low	Normal	Yes	No	
					40	Low	Normal	Yes	No	
					50	Low	Normal	Yes	No	
					60	Low	Normal	Yes	No	No

Disease Models & Mechanisms • Supplementary information

Table S4: DMSO dose-response observations. For each concentration of DMSO, 1 adult (older than 3 months) AB wildtype fish was placed in a glass beaker containing the drug and observed every 10 minutes, up to one hour. Behavior such as swim depth (whether the fish was swimming at the top, middle, or bottom of the glass beaker), swim rate, lack of movement, erratic movement, and survival after the hour dosing was observed.

Sex	Length (cm)	Weight (g)	Width (cm)	Dose (%)	Time (min)	Swim Depth	Swim Rate	Static Position	Erratic Movement	Survival After 60 min
Male	2.9	0.1986	0.5	0.010	0	Normal	Normal	No	No	
					10	Normal	Normal	No	No	
					20	Normal	Normal	No	No	
					30	Normal	Normal	No	No	
					40	Normal	Normal	No	No	
					50	Normal	Normal	No	No	
					60	Normal	Normal	No	No	Yes
Male	2.8	0.1527	0.5	0.030	0	Normal	Normal	No	No	
					10	Normal	Normal	No	No	
					20	Normal	Normal	No	No	
					30	Normal	Normal	No	No	
					40	Normal	Normal	No	No	
					50	Normal	Normal	No	No	
					60	Normal	Normal	No	No	Yes
Male	3.1	0.2569	0.6	0.060	0	Normal	Normal	No	No	
					10	Normal	Normal	No	No	
					20	Normal	Normal	No	No	
					30	Normal	Normal	No	No	
					40	Normal	Normal	No	No	
					50	Normal	Normal	No	No	
					60	Normal	Normal	No	No	Yes

Disease Models & Mechanisms • Supplementary information

Male	3.0	0.1847	0.5	0.090	0	Normal	Normal	No	No	
					10	Normal	Normal	No	No	
					20	Normal	Normal	No	No	
					30	Normal	Normal	No	No	
					40	Normal	Normal	No	No	
					50	Normal	Normal	No	No	
					60	Normal	Normal	No	No	Yes
Male	2.9	0.1744	0.5	0.30	0	Normal	Normal	No	No	
					10	Normal	Normal	No	No	
					20	Normal	Normal	No	No	
					30	Normal	Normal	No	No	
					40	Normal	Normal	No	No	
					50	Normal	Normal	No	No	
					60	Normal	Normal	No	No	Yes
Male	2.9	0.1553	0.5	0.50	0	Normal	Normal	No	No	
					10	Normal	Normal	No	No	
					20	Normal	Normal	No	No	
					30	Normal	Normal	No	No	
					40	Normal	Normal	No	No	
					50	Normal	Normal	No	No	
					60	Normal	Normal	No	No	Yes
Male	3.1	0.2117	0.5	1.0	0	Normal	Normal	No	No	
					10	Normal	Normal	No	No	
					20	Normal	Normal	No	No	
					30	Normal	Normal	No	No	
					40	Normal	Normal	No	No	
					50	Normal	Normal	No	No	
					60	Normal	Normal	No	No	Yes
Male	3.0	0.1629	0.5	1.5	0	Normal	Normal	No	No	
					10	Normal	Normal	No	No	
					20	Normal	Normal	No	No	

Disease Models & Mechanisms • Supplementary information

					30	Normal	Normal	No	No	
					40	Normal	Normal	No	No	
					50	Normal	Normal	No	No	
					60	Normal	Normal	No	No	No
Male	2.4	0.1334	0.5	2.0	0	Normal	Normal	No	No	
					10	Low	Slow	Yes	No	
					20	Low	Slow	Yes	No	
					30	Low	Slow/almost none	Yes	No	
					40	Low	Slow	Yes	No	
					50	Normal	Normal	Yes	No	
					60	Normal	Normal	No	No	No
Male	3.4	0.1861	0.5	2.5	0	Normal	Normal	No	No	
					10	Low	Slow	Yes	No	
					20	Near top	Slow	Minimal	No	
					30	At bottom or very top	Slow	Yes	No	
					40	At bottom or very top	Slow	Yes	Occasional darting to the top	
					50	Normal	Normal	Yes	No	
					60	Normal	Normal	No	No	No
